# Dual-Action Nucleic Acid Nanoparticles: Innate Immune Activation and Silver Nanocluster-Mediated Targeting of Intracellular Bacteria

**DOI:** 10.1021/acsnanomed.6c00069

**Published:** 2026-07-30

**Authors:** Leyla Danai, Christina J. Bayard, Krishna Majithia, Thomas Perrell, Phong Nguyen, Michael G. Walter, Alexander Lushnikov, Alexey V. Krasnoslobodtsev, M. Brittany Johnson, Yaroslava G. Yingling, Kirill A. Afonin

**Affiliations:** † Chemistry and Nanoscale Science Program, Department of Chemistry, 14727 University of North Carolina at Charlotte, Charlotte, North Carolina 28223, United States; ‡ Department of Materials Science and Engineering, 6798 North Carolina State University, Raleigh, North Carolina 27695, United States; § Department of Biology, University of North Carolina at Charlotte, Charlotte, North Carolina 28223, United States; ∥ Department of Physics, 14720 University of Nebraska Omaha, Omaha, Nebraska 68182, United States

**Keywords:** Silver nanoclusters, NANPs, RIG-I activation, antibacterial, *S. aureus*

## Abstract

Intracellular bacterial infections, such as those caused by *S. aureus*, are particularly challenging to treat because pathogens reside within host cells, evading immune defenses and limiting antibiotic efficacy, subsequently promoting drug resistance. To address this, we developed immunostimulatory nucleic acid nanoparticles (NANPs) functionalized with antibacterial silver nanoclusters to enable a dual mechanism of antibacterial action. These NANPs are engineered to activate RIG-I-mediated innate immune responses, which are often bypassed by intracellular bacteria to avoid host clearance. The embedded DNA-templated and -stabilized fluorescent silver nanoclusters, composed of approximately ten silver atoms, exhibit intrinsic antibacterial activity. We systematically evaluated different architectural configurations of silver nanoclusters within NANPs and assessed their relative stabilities, immunostimulatory potentials, and effects against planktonic bacteria and biofilms. We also confirmed that enhanced host immune activation and direct antibacterial activity result in a significant reduction of intracellular *S. aureus* in primary murine osteoblasts, highlighting the therapeutic potential of this platform.

## Graphical abstract

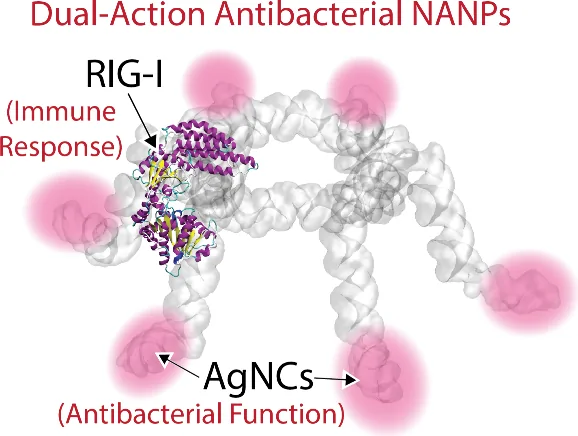

## Introduction

Osteomyelitis exemplifies the challenges of treating intracellular bacterial infections with nearly 80% of cases caused by *Staphylococcus aureus* (*S. aureus*). These infections are associated with prolonged illness, disability, and increased mortality.^1–4^ *S. aureus* employs multiple strategies to evade host defenses, including toxin production, biofilm formation, and, importantly, modulation of host immune responses. Critically, it can invade and proliferate within bone-resident cells such as osteoblasts, osteoclasts, and osteocytes.^4–6^ This intracellular niche protects *S. aureus* from immune detection and many conventional antibiotics which have limited penetration into host cells and thus, reduced activity against intracellular pathogens like *S. aureus*.^4,7^ As a result, infections frequently persist or recur, which highlights the need for new therapeutic strategies.

The rapid rise of antibiotic resistance further underscores the urgency for innovative antibacterial approaches.^8–11^ We present a strategy (Figure 1) that integrates an RNA nanoscaffold designed for controlled activation of the innate immune system with intracellular delivery of DNA-templated silver nanoclusters (DNA-AgNCs), potent antibacterial agents.^12–15^ This multipronged approach aims to enhance bacterial clearance while reducing the likelihood of resistance development.^16,17^

**Figure 1:**
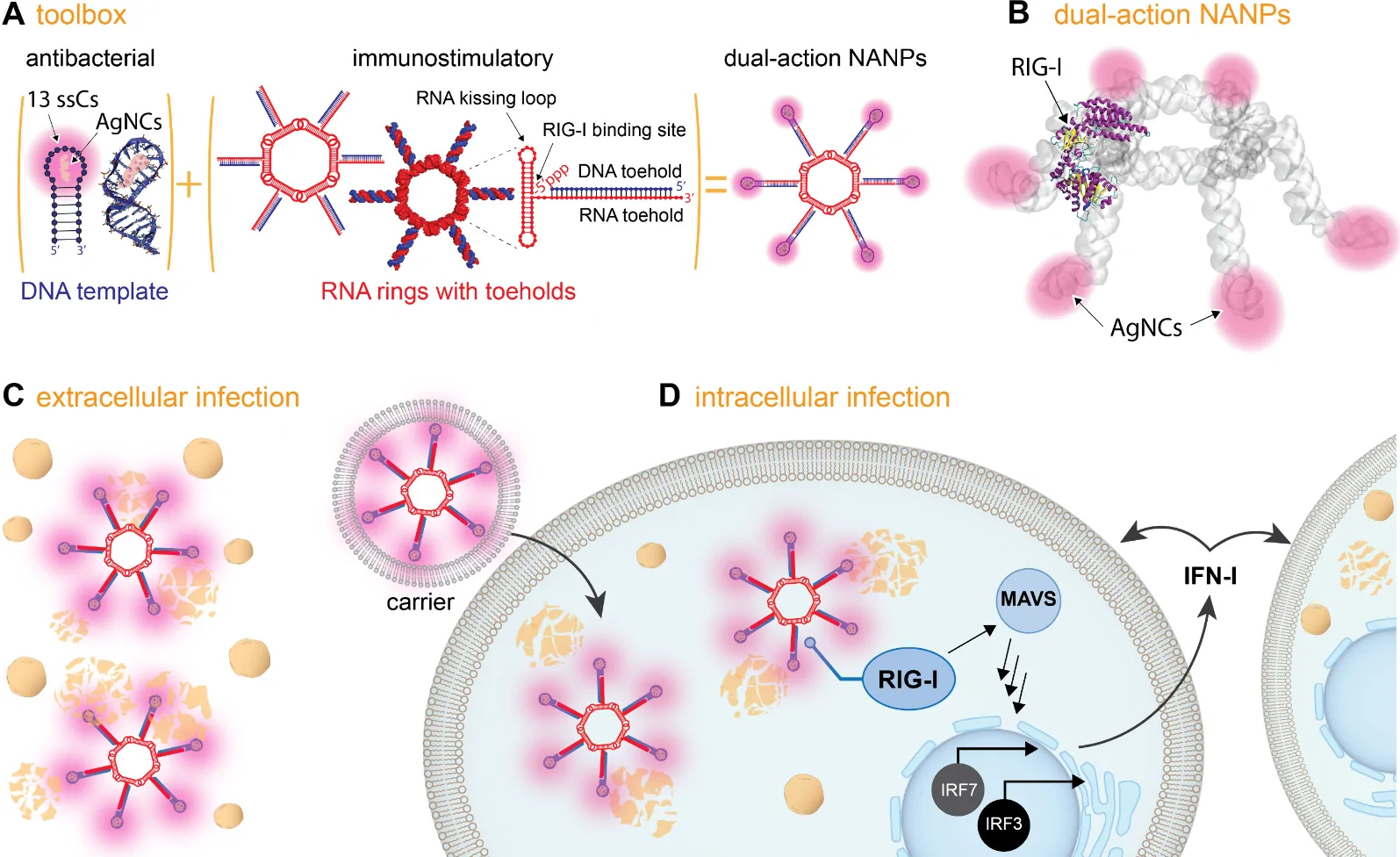
Dual-action nucleic acid nanoparticles (NANPs) are designed for simultaneous innate immune activation and silver nanocluster-mediated targeting of bacterial infections. (a) Schematic illustration of the design principles of immunostimulatory NANPs composed of RIG-I-activating RNA ring scaffolds decorated with antibacterial fluorescent silver nanoclusters (AgNCs). (b) Representative model of NANPs interacting with RIG-I. (c) AgNC rings promote extracellular bacterial killing. (d) Intracellular delivery of AgNC rings via Lipofectamine 2000 (L2K) promotes intracellular bacterial killing. Activation of RIG-I induces the production of type I interferons (IFNs), which signal to neighboring cells and enhance their resistance to bacterial infection.

DNA-AgNCs, first reported two decades ago,^18^ have been studied for their unique and modifiable fluorescent activities. Cytosine-rich, short single-stranded DNA sequences act as coordination sites for silver atoms, enabling the formation of sequence-defined fluorophores.^19^ DNA secondary structures further support AgNC templating, with structural stability closely correlated to fluorescence behavior.^19–23^ In this study, we employ a recently identified red-emitting DNA-AgNC sequence consisting of a hairpin with 13 single-stranded cytosines, which has demonstrated enhanced fluorescent stability and potent antibacterial activity against ESKAPE pathogens.^15^ This sequence is now further modified to enable its toehold-mediated integration into immunostimulatory nucleic acid nanoparticles (NANPs) for their simultaneous delivery to infected cells.

NANPs are highly adaptable agents with immunomodulatory properties that can be tailored to activate or suppress specific components of the innate immune system.^24–26^ Their modular design also enables incorporation of diverse therapeutic functionalities.^27,28^ We previously demonstrated that the spatial orientation of functional groups on hexagonal RNA ring-based NANPs can direct distinct innate immune responses through RIG-I activation.^29^ RIG-I is a cytosolic pattern recognition receptor that detects foreign nucleic acids by recognizing 5′-triphosphate groups on blunt-ended double-stranded RNA.^30,31^ While classically associated with antiviral defense;^30,32^ emerging evidence highlights its role in antibacterial immunity.^33–38^ Recent studies show that during *S. aureus* infection of bone-resident cells, RIG-I initiates protective interferon responses that limit intracellular bacterial burden, supporting its potential as a therapeutic target. Because RIG-I signaling is tightly regulated to prevent harmful hyperinflammation and cell death, its controlled activation is critical for developing therapies against acute and chronic bacterial infections.^17^

Small-angle X-ray scattering (SAXS) analysis of sugar pucker conformations in RNA rings indicates that exposure of 5′-triphosphate groups is enhanced when the rings incorporate six RNA functional groups.^39^ If RIG-I activation is primarily driven by the accessibility of these 5′-triphosphate moieties on ring-forming RNA strands, including those bearing toeholds, then the composition of the nucleic acid constructs attached to the toeholds should not significantly influence the RIG-I response. Accordingly, RNA rings functionalized with six hairpin DNAs are expected to retain the capacity to activate RIG-I, while simultaneously enabling additional therapeutic functionality through the attached moieties.

Current approaches for treating intracellular bacterial infections by harnessing innate immune responses include antimicrobial peptides and host-directed therapies to activate macrophages and suppress intracellular bacterial survival.^40,41^ Dual-functional therapies, such as nanoparticles comprising both an antimicrobial peptide and an antibiotic, are reported for reaching intracellular bacteria within host cells.^42^ Here, we investigate immunostimulatory RNA rings functionalized with DNA-AgNCs as a novel dual-action antibacterial platform. Although DNA-AgNCs and immunostimulatory RNA rings have each been studied independently, their integration into a single nanoscale construct represents an innovative strategy that combines direct silver-mediated antibacterial activity with host-directed immune stimulation. This dual mechanism enables simultaneous targeting of pathogens and activation of RIG-I-mediated innate immune responses, offering a fundamentally different approach to conventional antibiotics. Moreover, the programmable architecture NANPs allows precise control over AgNC loading, spatial organization, and immunostimulatory potency, creating a modular platform that can be readily optimized and adapted to combat intracellular and drug-resistant bacterial infections. The resulting constructs (Figure 2a and SI Figure S1) were designed with six outward-facing AgNCs (6 OUT), six inward-facing AgNCs (6 IN), or 12 AgNCs in both orientations (12 IN-OUT) and evaluated for AgNC stability, immune activation, and antibacterial efficacy. By combining direct bacterial killing with immune stimulation, this platform enhances host defenses, reduces intracellular bacterial burden, and may help limit the emergence of antimicrobial resistance.^43^

**Figure 2:**
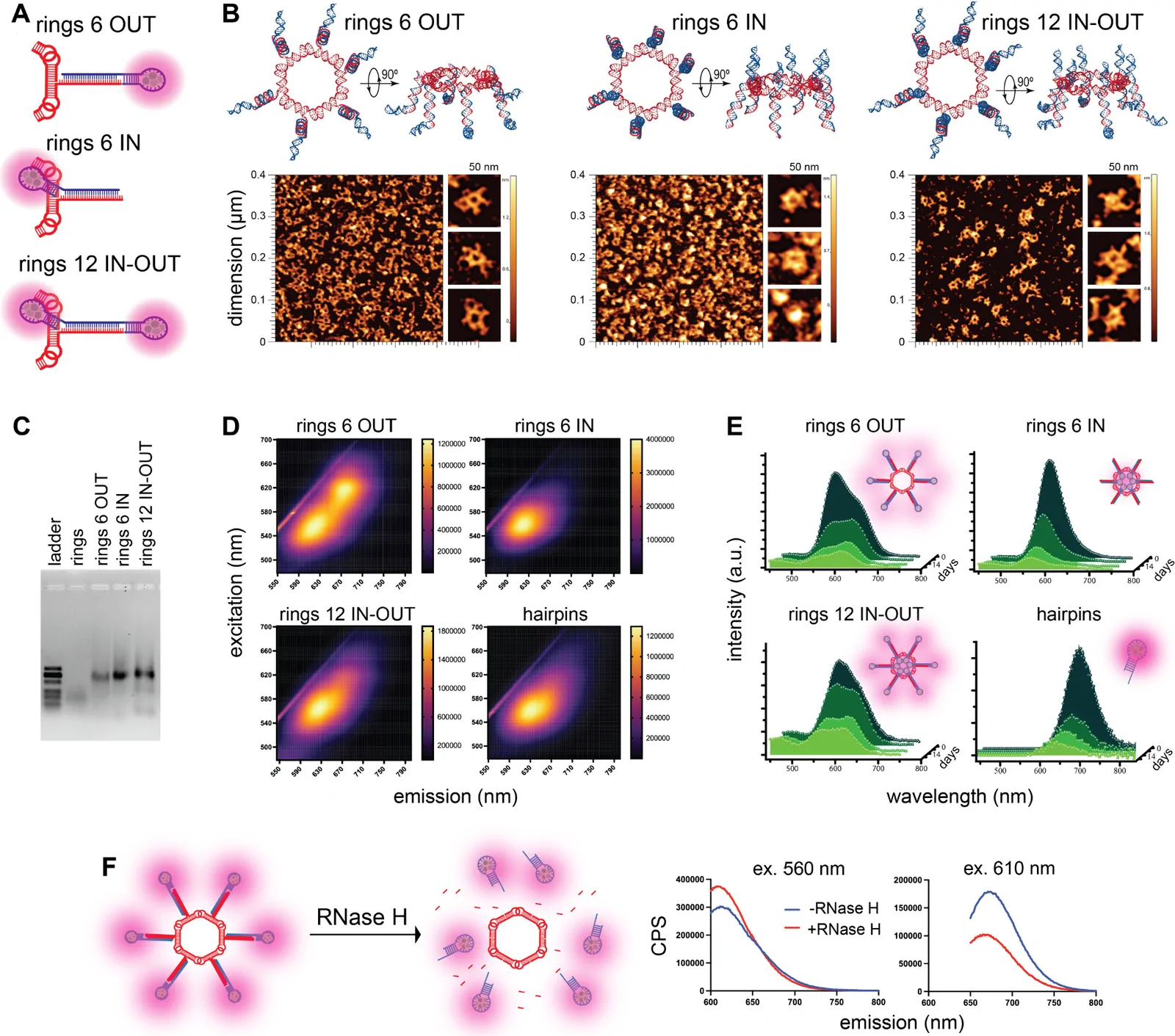
Structural and fluorescent characterization of DNA-AgNC-functionalized RNA rings. (a) Schematic representation of different orientations of antibacterial AgNCs within immunostimulatory RNA ring scaffolds (only one extension is shown). (b) 3D models with corresponding representative atomic force microscopy (AFM) images. (c) 1.5% native agarose gel analysis of the assembled structures. (d) 3D excitation–emission fluorescence scans and (e) fluorescence aging profiles of RNA rings. (f) RNase treatment of Ring 6 OUT releases AgNC-containing hairpins and alters their fluorescence emission intensities.

## Material and Methods

All materials and methods are detailed in the Supporting Information.

### Synthesis of Dual-Action NANPs

RNA monomers were synthesized by in vitro runoff transcription from PCR-amplified DNA templates using T7 RNA polymerase. Purified RNA strands were annealed under controlled salt and temperature conditions to form RNA rings, which were confirmed by EMSA and stored at 4 °C until use. *DNA-AgNCs* were synthesized by mixing constructs with AgNO_3_ followed by reduction with freshly prepared NaBH_4_.

### Atomic Force Microscopy (AFM)

All NANPs were deposited on APS-modified mica, washed, and dried under argon prior to imaging. AFM imaging was performed in PeakForce tapping mode and analyzed by using Gwyddion software.

Fluorescence emission spectra were recorded by using a FluoroMax Plus spectrofluorometer. Samples were excited at 530 nm, and emission was collected at 636 nm under standardized slit conditions.

3D excitation–emission scans were collected using a FluoroMax Plus spectrofluorometer over defined excitation and emission ranges, and spectral maps were generated for comparative fluorescence analysis.

Fluorescence aging was evaluated using excitation–emission matrices at multiple time points over 21 days.

### Energy-Dispersive X-ray Spectroscopy (EDS)

Samples were deposited onto boron substrates and analyzed using SEM-EDS after complete drying for elemental composition to estimate silver content within the DNA-AgNCs.

Singlet oxygen generation was measured using infrared emission spectroscopy upon 565 nm excitation. Emission between 1200 and 1500 nm was integrated to quantify reactive oxygen species production.

### Molecular Dynamics Simulations

Coarse-grained oxDNA- and ANM-based simulations were carried out to model NANP interactions with RIG-I.

Immune reporter cell assays used HEK Lucia RIG-I reporter cells, and RIG-I activation was quantified by luminescence readout.

### Culture and Maintenance of Primary Murine Osteoblasts

Cells were isolated from neonatal mouse calvaria and cultured under osteogenic differentiation conditions. All animal procedures were conducted under protocol IACUC-26-032, “Breeding Protocol for the Isolation of Bone and Brain Cells”.

*S. aureus* UAMS-1 was cultured in LB medium under standard conditions, and bacterial density was quantified spectrophotometrically.

MIC and MBC were estimated by treating bacteria with various constructs followed by growth assessment and colony counting. The MBC was defined as 3-log reduction (≥99.9%) reduction in viability.

Biofilm formation was quantified using crystal violet staining following incubation with constructs and compared to that of untreated controls.

### Scanning Electron Microscopy (SEM)

*S. aureus* samples were fixed, dehydrated, sputter-coated, and imaged by SEM.

*S. aureus* infection of primary murine osteoblasts was done at defined MOI under antibiotic-free conditions. After infection, extracellular bacteria were eliminated, and intracellular responses were analyzed.

### Transfection of Primary Murine Osteoblasts

Infected osteoblasts were transfected with constructs or AgNC constructs by using commercial delivery agents. Posttreatment bacterial survival was quantified via CFU counting.

### Immunofluorescent Microscopy

Transfected osteoblasts were fixed and stained for actin and nuclei to visualize cellular morphology, and imaging was performed using confocal microscopy.

### ELISA

IL-6 and IFN-β production was quantified using sandwich ELISA following infection and treatment and standard curves were used to calculate the concentrations.

### Immunoblot Analysis

RIG-I protein expression was assessed by Western blotting using specific antibodies, with β-actin as a loading control.

### Statistical Analysis

Data were analyzed using GraphPad Prism and presented as mean ± SD or SEM as indicated. Statistical significance was defined as *p* < 0.05 with corrsponding tests specified in figure legends.

## Results and Discussion

### Structure and Fluorescence of DNA-AgNC-Functionalized RNA Rings

All assemblies were validated using MD simulations, AFM, and EMSA (Figure 2b, c). Each RNA ring was confirmed to contain six extensions consistent with the designed architecture. The most distinct structural features were observed for rings 6 OUT, in which all DNA-AgNC extensions are readily visible due to their outward orientation in the imaging plane. In contrast, rings 6 IN are not coplanar with the ring scaffold; steric constraints position the extensions above or below the plane of the ring, sometimes partially or completely filling the central cavity, which appears reduced in AFM images. The Ring 12 IN-OUT variant displays a combination of these features: outward-facing extensions are clearly distinguishable, as in 6 OUT, while some extensions occupy positions above or below the ring plane, producing a partially occluded central opening characteristic of 6 IN.

Following the assembly of DNA-AgNCs on the RNA ring scaffolds, 3D emission scans were performed to compare the hairpin DNA-AgNCs and the functionalized ring structures (Figure 2d), with 2D emission spectra collected for reference (SI Figure S2). Unbound hairpins exhibit maximum emission at 636 nm with 568 nm excitation. Rings 6 IN show a slight red-shift, with excitation and emission maxima at 560 and 621 nm, respectively. Interestingly, rings 6 OUT display two distinct emission peaks: one at 626 nm, similar to the hairpins and rings 6 IN, and a second at 674 nm. Previous studies indicate that cytosine loops of hairpin AgNCs can dimerize intermolecularly, forming distinct AgNC species with altered absorption and emission profiles.^44,45^ It is possible that outward-facing hairpin AgNCs on ring 6 OUT interact with AgNCs on neighboring rings, although further studies are needed to elucidate this effect. Energy-dispersive X-ray spectroscopy confirmed that each hairpin contains about 10 silver atoms (SI Figure S3 and Table S1).^19^ The fluorescence quantum yields of rings 6 OUT, rings 6 IN, rings 12 IN-OUT, and the free hairpin were all calculated at equimolar silver (SI Table S2). Rings 12 IN-OUT are associated with the highest quantum yield of 4.52%, significantly higher than the other three structures.

Fluorescence of the assemblies was monitored over 21 days (Figure 2e). Although fluorescence gradually decreased over time, these measurements demonstrate the relative stability of the DNA-AgNCs scaffolded on different nanostructures and allow direct comparison with free hairpins. All three ring structures exhibited greater long-term fluorescence stability than the free hairpins, highlighting the stabilizing effect of the RNA scaffold on the DNA-AgNCs. Rings 12 IN-OUT retained the highest fluorescence intensity over 14 days, while Rings 6 OUT and 6 IN lost fluorescence intensity comparable to the free hairpins. The second emission maxima observed with Rings 6 OUT and Rings 12 IN-OUT corresponding to approximately λ_EM_ = 675 nm (with λ_EXC_ = 615 nm) persists throughout overall loss of fluorescence intensity. To determine whether the 610 nm emission peak is specific to the Ring 6 OUT structure, RNase H was used to selectively degrade the RNA strands bound to DNA, thereby releasing the AgNC hairpins from the ring scaffold (Figure 2f). Following RNase H treatment, the emission peak at 560 nm, characteristic of free hairpins, increased in intensity, while the 610 nm peak simultaneously decreased. These complementary shifts in emission confirm that the 610 nm peak is uniquely associated with the Ring 6 OUT architecture.

### Bactericidal Properties of DNA-AgNC-Functionalized RNA Rings

The minimum inhibitory concentration (MIC) and minimum bactericidal concentration (MBC) of hairpin DNA-AgNCs against S. aureus were determined to assess whether the AgNCs act bacteriostatically or bactericidally. The MIC was 6 μM DNA (78 μM silver), the concentration at which no visible growth or change in optical density (OD) was observed after 18 h (SI Figure S4). Colony counts indicated an MBC of approximately 6 μM DNA, reduction in bacterial viability (Figure 3a). Because the MBC/MIC ratio is less than 4, the DNA-AgNCs are classified as bactericidal rather than bacteriostatic.^46^ At the same DNA-AgNC concentration, Rings 6 OUT, 6 IN, and 12 IN-OUT also demonstrated potent antibacterial activity, reducing the initial bacterial population by more than 3 logs. Sytox staining, dependent on membrane integrity, indicates that AgNCs kill bacteria by compromising cell walls and membranes (Figure 3b). The AgNCs were also effective at inhibiting *S. aureus* biofilm formation for at least 18 h, as evidenced by the reduced biofilm density visualized in crystal violet-stained treated samples compared to buffer-treated controls (Figure 3c). SEM images of *S. aureus* show that treatment induces pronounced cell damage and swelling, whereas buffer-treated controls exhibit no morphological alterations, with smooth and intact bacterial cell surfaces (Figure 3d). Additional representative SEM images are shown in the SI Figure S5 to further support these observations.

**Figure 3:**
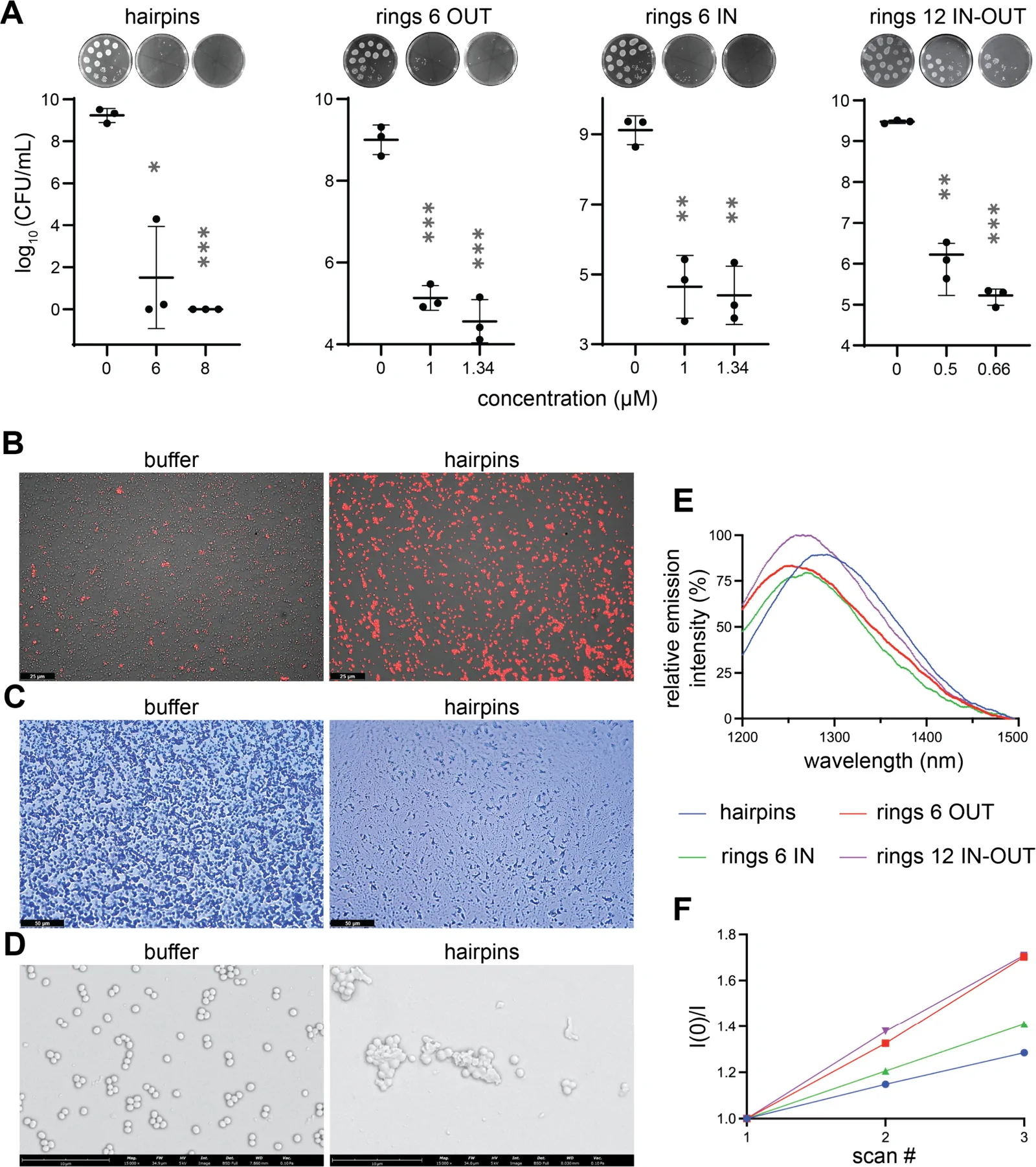
Antibacterial activity and mechanistic studies of DNA-AgNC assemblies. (a) *S. aureus* colony counts for minimum bactericidal concentration (MBC) determination of hairpins and RNA-AgNC rings (6 OUT, 6 IN, and 12 IN–OUT). Representative images of bacterial colonies on agar plates are shown above. Asterisks indicate statistical significance compared to 0 μM, determined by Welch’s *t* test (p values: * ≤ 0.05, ** ≤ 0.01, *** ≤ 0.001, **** ≤ 0.0001). (b) *S. aureus* biofilms stained with Sytox Deep Red after treatment with buffer or 6 μM hairpins. (c) *S. aureus* biofilm formation after 24 h incubation of planktonic cells with buffer or 6 μM hairpins, followed by crystal violet staining. (d) SEM images of *S. aureus* following 6 μM DNA-AgNC hairpin treatment. (e) Singlet oxygen emission of RNA-AgNC rings upon excitation at 565 nm. (f) Degradation of AgNC rings and hairpins following singlet oxygen emission.

While the antibacterial mechanisms of silver nanoparticles are well-established, analogous studies for DNA-AgNCs are limited. Silver nanoparticles often exert toxicity via the production of reactive oxygen species including singlet oxygen. To determine whether DNA-AgNCs induce oxidative stress in bacteria, singlet oxygen generation was measured for each structure following excitation at 565 nm. All AgNC-functionalized rings and free hairpins produced singlet oxygen (Figure 3e). The highest emission intensity was observed for rings 12 IN-OUT, whereas rings 6 IN generated approximately 75% of this maximum. Notably, rings 12 IN-OUT degraded most rapidly under singlet oxygen exposure, while rings 6 IN and free hairpins were more stable (Figure 3f). The enhanced stability of rings 6 IN, comparable to that of free hairpins, may result in part from the inward orientation of the AgNC-templating hairpins, which reduces their exposure to oxidative degradation.

### DNA-AgNC-Functionalized RNA Rings Trigger RIG-I-Mediated Immune Responses

Molecular dynamics simulations were performed to investigate interactions between RIG-I and Ring 6 OUT. Coarse-grained oxDNA simulations (Figure 4a) were complemented with all-atom models (Figure 4b), both showing a single RIG-I protein binding to the 5′-triphosphates at the nick of individual RNA ring monomers. A partial side view of the all-atom model highlights the RIG-I binding site on a single RNA monomer (Figure 4c). All-atom models of Ring 6 IN and Ring 12 IN-OUT are provided in the SI Figure S6. Applying a mutual trap force between ANM-oxDNA protein residues and the DNA-AgNC-functionalized RNA rings proved overly restrictive, confining the protein unless trap stiffness was reduced to unrealistically low values. Stability comparisons across the three systems were therefore based on potential energy (PE) alone. Ring 12 IN-OUT exhibited the lowest PE (SI Figure S7), likely due to higher effective system density. Ring 6 OUT consistently showed lower PE than Ring 6 IN (SI Figures S7, S8; *p* < 0.05), suggesting greater stability under these conditions, although additional trajectories are needed to confirm this trend. Ring 6 IN showed a larger mean |ΔPE| compared to Ring 6 OUT (0.00743 vs 0.00517), but the difference was not statistically significant (Welch *t* test p = 0.092).

**Figure 4:**
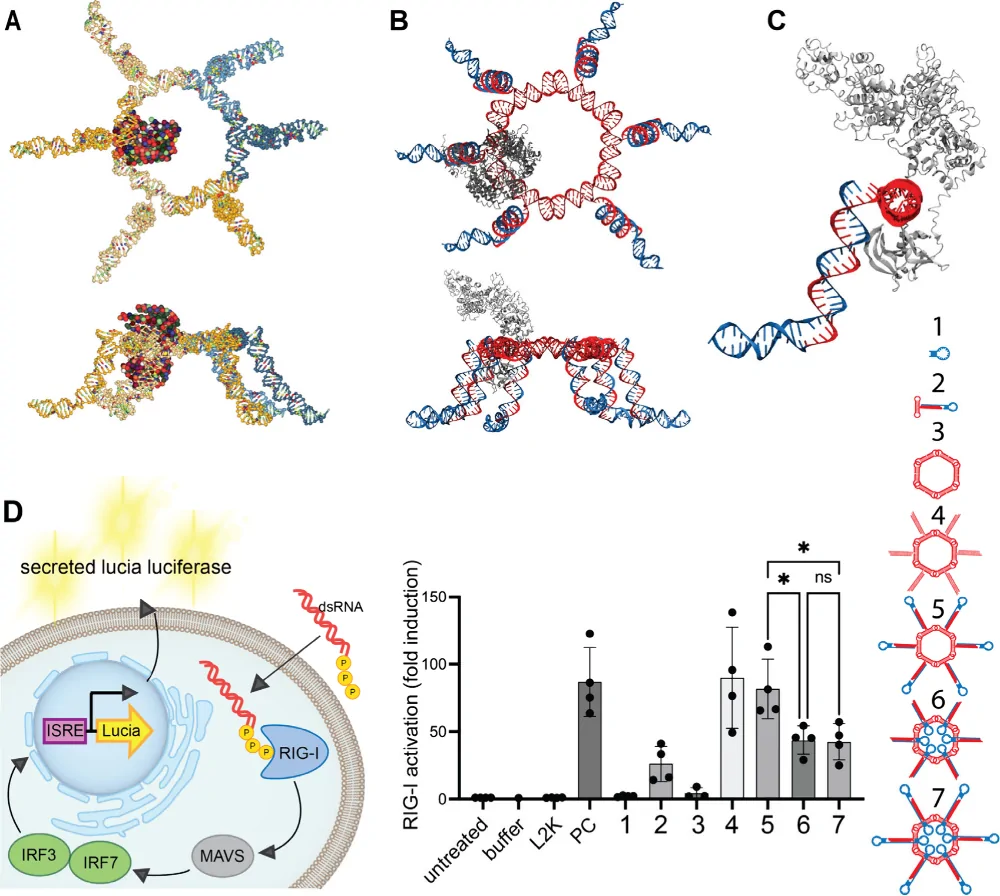
DNA-AgNC-functionalized RNA rings trigger RIG-I-mediated immune activation. (a) oxDNA models of RIG-I protein binding interactions with Ring 6 OUT. (b) All-atom models of RIG-I protein binding interactions with Ring 6 OUT. (c) Partial side view of Ring 6 OUT monomer bound to RIG-I. Blue is associated with DNA, and red is RNA. (C) Luciferase detection assay for measuring relative RIG-I activity. (d) RIG-I activation in HEK Lucia RIG-I cells after 24 h of treatment with various constructs schematically shown on the right. Data represent three biological replicates, normalized to the negative control of untreated cells. Statistical significance was determined using ordinary one-way ANOVA. Negative controls included Lipofectamine 2000 (L2K), buffer (100 mM NH_4_OAc, 2 mM Mg^2+^), and untreated cells. As a positive control, 5′-triphosphate single-stranded RNA (3P) was included to confirm RIG-I stimulation.

An all-atom duck RIG-I model (PDB ID: 4A2W), completed with MODELER and AlphaFold, was manually positioned relative to the nanorings to approximate the orientation reported in experimental structural studies.^47^ This configuration was used to visually assess potential steric clashes associated with single-RIG-I binding. In the initial ring 6 IN and ring 12 IN-OUT models, the DNA hairpin loop at the top of the construct exhibited substantial steric clashes with the RIG-I model, which impeded wrapping of RIG-I around the nanoring. The adjusted conformation places RIG-I closer to the center of the ring than that in the Ring 6 OUT model.

To assess relative RIG-I activation by the AgNC-functionalized RNA rings, human embryonic kidney (HEK) Lucia RIG-I reporter cells were transfected with rings 6 OUT, rings 6 IN, and rings 12 IN-OUT. RNA rings with six double-stranded RNA extensions, previously shown to stimulate RIG-I, were included as a positive control.^29^ RIG-I activation was measured by using a luciferase detection assay (Figure 4b). Notably, rings 6 OUT elicited higher RIG-I activation compared to rings 6 IN and rings 12 IN-OUT (Figure 4c), suggesting that the outward orientation of the hairpins may facilitate more accessible and efficient interactions with RIG-I.

### DNA-AgNC-Functionalized RNA Rings Induce Antibacterial and RIG-I-Dependent Cytokine Responses against Intracellular *S. aureus* in Primary Osteoblasts

We have previously demonstrated that free DNA-AgNC structures inhibit planktonic bacterial growth for several hours.^14,15^ Notably, we have documented that RIG-I-dependent responses are protective during *S. aureus* infection of bone cells and contribute to the restriction of intracellular bacterial burden.^48,49^ Based on these findings, we hypothesized that coupling DNA-AgNCs to immunostimulatory RNA rings, specifically Rings 6 OUT, identified as the most potent RIG-I agonist (Figure 4d), would provide a dual therapeutic advantage: direct antibacterial activity from the AgNCs and enhanced protective host defenses mediated by RIG-I. To evaluate intracellular compatibility, these constructs were delivered into primary murine osteoblasts. Immunofluorescence microscopy confirmed efficient intracellular uptake of both free hairpins (HP) and ring constructs containing six extensions, as well as a single DNA-AgNC hairpin in the outward orientation, referred to as Ring-HP. (Figures 5a, b). Notably, osteoblasts express low constitutive levels of RIG-I that are further upregulated specifically in response to the Ring-HP construct and positive control ligand, 5′ triphosphate RNA (3P), but not the HP alone (Figure 5c and SI Figure S9a). Accompanying changes in RIG-I protein expression, osteoblasts produce the proinflammatory cytokine interleukin-6 (IL-6) in response to both constructs (Figure 5d); however, IFN-β was produced in response to only the Ring-HP construct and not the single HP (Figure 5d). Such responses to the Ring-HP construct and control ligand 5′ triphosphate RNA (3P) are RIG-I dependent as siRNA mediated knockdown of RIG-I expression results in a significant decrease in osteoblast production of IL-6 and IFN-β (Figure 5d and SI Figure S9b). This further supports the RIG-I agonist activity observed for the RING constructs in reporter cell lines.

**Figure 5:**
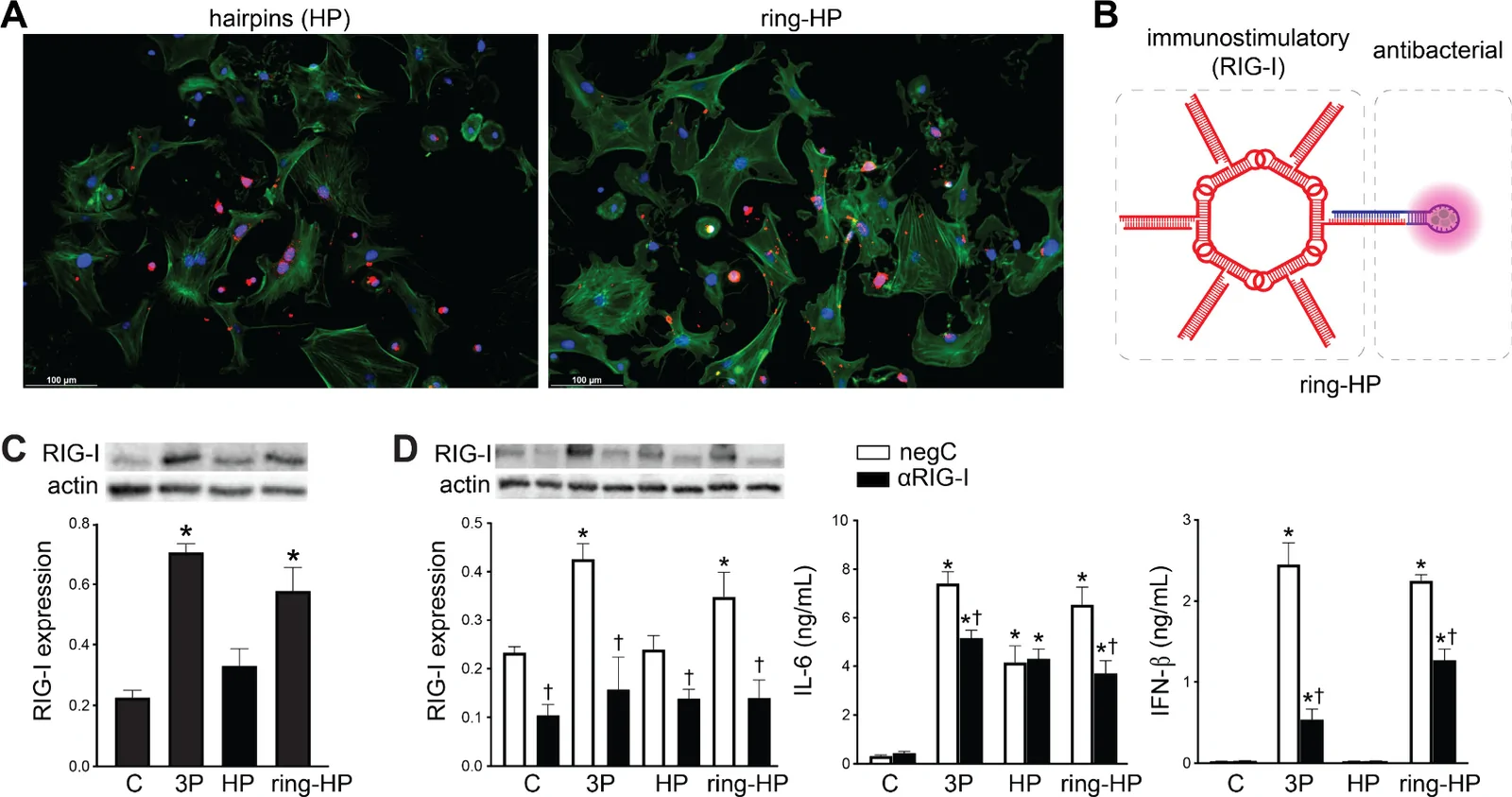
DNA-AgNC-functionalized RNA rings induce RIG-I-dependent responses in *S. aureus*-infected osteoblasts. Primary murine osteoblasts were unstimulated or stimulated with 5′triphosphate RNA (3P; 5 μg/mL), single HP (13 μM silver), or RNA rings containing six extensions, as well as a single DNA-AgNC hairpin in the outward orientation, referred to as Ring-HP (13 μM silver) using Lipofectamine 2000. (a) Immunofluorescent microscopy of osteoblasts 4 h post-transfection, AgNC rings are shown in red, and the actin cytoskeleton of the osteoblasts appears green. (b) Schematic structure of Ring-HP. (c) RIG-I expression at 24 h post-transfection assessed by immunoblot analysis. Primary murine osteoblasts were transfected with siRNA (15 nM) directed against RIG-I or negative control siRNA (NegC) using RNAiMAX prior to stimulation with 3P, HP, and Ring-HP. (d) RIG-I expression, IL-6 production, and IFN-β production by osteoblasts at 24 h post-transfection, respectively. Representative blots and densitometric quantification of RIG-I expression normalized to β-actin levels. Asterisks indicate a statistically significant increase compared to the corresponding unstimulated control; daggers represent a statistically significant reduction compared to the similarly treated NegC (mean ± SEM, *n* = 3, two-way ANOVA, p value <0.05).

Next, to evaluate the ability of HP, RNA rings containing six dsRNAs (referred to as Ring), and Ring-HP constructs to stimulate protective immune responses and control bacterial burden, we infected primary murine osteoblasts with *S. aureus* in the absence and presence of these constructs (Figure 6a). Consistent with previous studies,^48,50^ *S. aureus* infection alone increased IL-6 and IFN-β production by osteoblasts (Figure 6b). As expected, IFN-β levels were further elevated in the presence of recombinant IFN-β and Ring-HP, but not the HP alone. Excitingly, both the HP and Ring-HP significantly reduced intracellular bacterial burden in infected osteoblasts, along with recombinant IFN-β as previously shown^51^ (Figure 6c), without impacting host cell viability (Figure 6d). As recombinant IFN-β alone reduced intracellular *S. aureus* burden (Figure 6c), these findings suggest that the antibacterial activity observed with the Ring-HP likely reflects both direct AgNC-mediated killing and RIG-I-dependent antibacterial responses. Importantly, while the HP alone functions through direct antibacterial activity, the Ring-HP construct combines both direct antibacterial effects and RIG-I-mediated immunostimulatory activity, promoting a dual mechanism for controlling intracellular bacterial burden.

**Figure 6:**
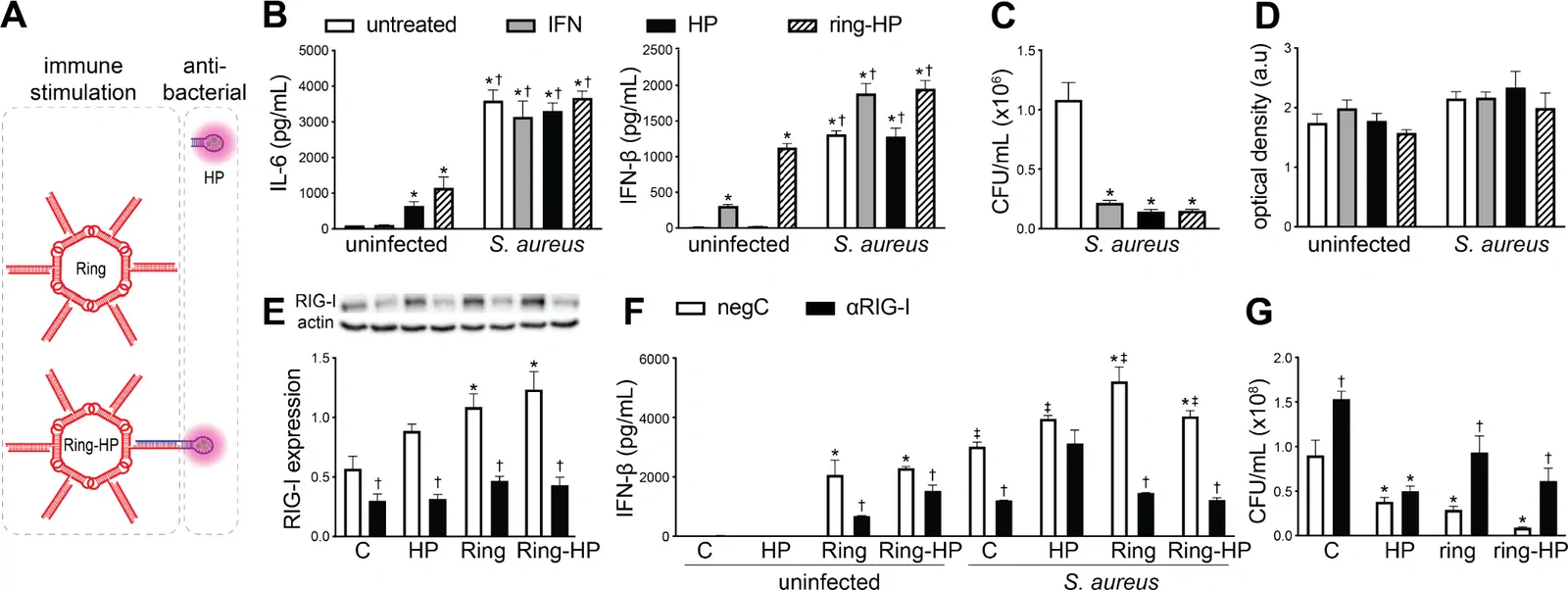
DNA-AgNC-functionalized RNA rings induce antibacterial RIG-I-dependent responses in *S. aureus*-infected osteoblasts. (a) Schematic structures of HP, Ring, and Ring-HP constructs. Primary murine osteoblasts were uninfected or infected with *S. aureus* (MOI 75:1) prior to treatment with IFN-β (IFN; 1 ng/mL), or transfection of HP (13 μM silver) or Ring-HP (13 μM silver) using Lipofectamine 2000. (b) IL-6 and IFN-β production by osteoblasts at 24 h, respectively. (c) Viable intracellular *S. aureus* quantified by colony counting at 24 h. (d) Osteoblast viability assessed by MTS at 24h. Asterisks indicate statistically significant differences compared to the untreated control; daggers represent a significant increase compared to the corresponding uninfected control (mean ± SEM, *n* = 3, two-way ANOVA (b, d) and one-way ANOVA (c), p value < 0.05). Primary murine osteoblasts were transfected with siRNA (15 nM) directed against RIG-I or negative control siRNA (NegC) using RNAiMAX. Cells were then uninfected or infected with *S. aureus* (MOI 75:1) prior to transfection of HP (13 μM silver), Ring (0 μM silver) or Ring-HP (13 μM silver) using Lipofectamine 2000. (e) RIG-I expression at 24 h post-transfection assessed by immunoblot analysis. Representative blots and densitometric quantification normalized to β-actin levels. (f) IFN-β production by osteoblasts at 24 h post-transfection. (g) Viable intracellular *S. aureus* quantified by colony counting at 24 h. Asterisks indicate a significant difference compared to the corresponding untreated control; daggers represent a significant difference compared to the similarly treated NegC control; double daggers represent statistical significance compared to the uninfected group (mean ± SEM, *n* = 3, two-way ANOVA, p value < 0.05).

To confirm that RNA ring-mediated responses were RIG-I-dependent, an siRNA approach was used to knockdown RIG-I expression prior to stimulation with HP, Ring, and Ring-HP (Figure 6e and SI Figure S9c) and infection with *S. aureus*. RIG-I knockdown markedly reduced RIG-I expression levels across all treatment groups. Additionally, RIG-I knockdown substantially reduced IFN-β production in response to the Ring and Ring-HP, supporting RIG-I agonist activity (Figure 6f). As anticipated, IFN-β production in response to the HP alone was not impacted by RIG-I knockdown (Figure 6f). In agreement with our previous studies noting a protective role for type I IFNs in restricting bacterial burden inside osteoblasts,^42,45^ reduced production of IFN-β corresponded with an increase in bacterial burden (Figure 6g). As such, delivery of the HP significantly reduced bacterial burden compared to the control group but was not affected by RIG-I knockdown consistent with the direct antibacterial activity of this construct. In contrast, delivery of both the Ring and the Ring-HP significantly reduced intracellular bacterial burden compared to the control group (Figure 6g). However, RIG-I knockdown significantly reduced osteoblast production of IFN-β in response to these constructs resulting in an overall increase in bacterial burden. Notably, the Ring-HP retained some antibacterial activity following RIG-I knockdown, due to the direct antibacterial activity associated with the HP (Figure 6g). These findings indicate that RIG-I-dependent responses contribute to the observed reduction in bacterial burden and that these protective responses can be further enhanced by the inclusion of direct antibacterial HPs. Together, these effects provide a promising foundation for developing therapeutics against intracellular pathogens.

## Conclusion

The coalescence of antibacterial silver nanoclusters paired with immunostimulatory RNA scaffolds allows for combating intracellular *S. aureus* infections in vitro from two different angles. As the immunostimulatory properties of functionalized RNA rings are dependent on monomer orientation rather than exclusively composition, DNA extensions with silver nanoclusters can be included without hampering immunostimulation. This therapeutic formulation is effective against intracellular bacteria and introduces the dual effect of silver nanoclusters and RNA nanotechnology to destroy invasive pathogens and enhance immune activation of infected host cells.

## Supplementary Material



## Data Availability

The data supporting the findings of this study are available from the corresponding author upon reasonable request.

## Notes

Nucleic acid sequences used in this study; schematic of assembly (Figure S1); additional physicochemical characterization of assemblies their biological activity, simulations, and experiments (Figures S2–S9). EDS elemental composition (Table S1). (PDF)
