# Supplementary material for: Dual-Action Nucleic Acid Nanoparticles: Innate Immune Activation and Silver Nanocluster-Mediated Targeting of Intracellular Bacteria
Source: ACS Nano Med. 2026 Jul 30;1(9):2406–15. doi: 10.1021/acsnanomed.6c00069 (PMC13492571; doi:10.1021/acsnanomed.6c00069)
Supplement: Supplementary file 1 [file nm6c00069_si_001.pdf]

Supporting Information

Dual-Action Nucleic Acid Nanoparticles: Innate Immune Activation and Silver Nanocluster-Mediated Targeting of Intracellular Bacteria

Leyla Danai[1], Christina J. Bayard[2], Krishna Majithia[3], Thomas Perrell[1], Phong Nguyen[1], Michael G. Walter[1], Alexander Lushnikov[4], Alexey V. Krasnoslobodtsev[4], M. Brittany Johnson[3], Yaroslava G. Yingling[2], Kirill A. Afonin[1]*

1 – Chemistry and Nanoscale Science Program, Department of Chemistry, University of North Carolina at Charlotte, Charlotte, NC 28223, USA
2 – Department of Materials Science and Engineering, North Carolina State University, Raleigh, NC 27695, USA
3 – Department of Biology, University of North Carolina at Charlotte, Charlotte, NC 28223, USA
4 – Department of Physics, University of Nebraska Omaha, Omaha, NE 68182, USA

* correspondence to Kirill A. Afonin at kafonin@charlotte.edu

Sequences used in this project (5' → 3')

RNA ring scaffold:

Strand A:
GGGAACCGUCCACUGGUUCCCGCUACGAGAGCCUGCCUCGUAGCUUCGGUGGUGCAGA
UGAACUUCAGGGUCA

Strand B:
GGGAACCGCAGGCUGGUUCCCGCUACGAGAGAACGCCUCGUAGCUUCGGUGGUGCAGA
UGAACUUCAGGGUCA

Strand C:
GGGAACCGCGUUCUGGUUCCCGCUACGAGACGUCUCCUCGUAGCUUCGGUGGUGCAGA
UGAACUUCAGGGUCA

Strand D:
GGGAACCGAGACGUGGUUCCCGCUACGAGUCGUGGUCUCGUAGCUUCGGUGGUGCAG
AUGAACUUCAGGGUCA

Strand E:
GGGAACCACCACGAGGUUCCCGCUACGAGAACCAUCCUCGUAGCUUCGGUGGUGCAGA
UGAACUUCAGGGUCA

Strand F:
GGGAACCGAUGGUUGGUUCCCGCUACGAGAGUGGACCUCGUAGCUUCGGUGGUGCAG
AUGAACUUCAGGGUCA

Hairpin DNA:
TATCCGTCCCCCCCCCCCCCACGGATA

Hairpin DNA for Rings 6-OUT:
TATCCGTCCCCCCCCCCCCCACGGATAACCCTGAAGTTCATCTGCACCACCG

Hairpin DNA for Rings 6-IN:
ACCCTGAAGTTCATCTGCACCACCGTATCCGTCCCCCCCCCCCCCACGGATA

Hairpin DNA for Rings 12 IN-OUT:
TATCCGTCCCCCCCCCCCCCACGGATAACCCTGAAGTTCATCTGCACCACCGTATCCGTC
CCCCCCCCCCCACGGATA

RIG-I agonist "3P":
pppGGAGCAAAAGCAGGGUGACAAAGACAUAAUGGAUCCAAACACUGUGUCAAGCUUUCA
GGUAGAUUGCUUUCUUUGGCAUGUCCGCAAAC

"Rings 6 RNA" extension:
pACCCUGAAGUUCAUCUGCACCACCG

Materials and Methods

Synthesis of functional RNA rings. RNA ring monomers were synthesized in the laboratory by *in vitro* transcription (IVT), while all DNA monomers were purchased from Integrated DNA Technologies (IDT). Run-off IVT was performed using PCR-amplified DNA templates and T7 RNA polymerase under optimized reaction conditions (80 mM HEPES-KOH, pH 7.5; 2.5 mM spermidine; 50 mM DTT; 2 mM $MgCl_2$; 25 mM rNTPs; 0.2 μM DNA template; ~100 U/μL T7 RNA polymerase) for 3.5 h at 37 °C. Following transcription, reactions were treated with RQ1 RNase-free DNase (Promega) for 30 min at 37 °C to remove DNA templates. RNA products were purified by 15% denaturing PAGE (8 M urea), visualized under UV light, excised, and eluted overnight using crush-and-soak buffer, followed by ethanol precipitation and washing to obtain highly purified RNA monomers. RNA rings were assembled in a one-pot reaction by mixing the corresponding RNA and DNA monomers in nuclease-free water, followed by heating to 95 °C for 2 min and snap-cooling on ice for 2 min. Assembly was completed by adding ammonium acetate buffer (100 mM NH_4OAc, 2 mM $MgCl_2$, pH 6.9) and incubating for 30 min at 30 °C, with formation confirmed by EMSA and products stored at 4 °C until use.

Production of DNA-AgNCs. DNA-templated silver nanoclusters (DNA-AgNCs) were synthesized by mixing $AgNO_3$ with the specified DNA constructs in 4 mM NH_4OAc (pH 6.9) at a 1:1 Ag^+:cytosine ratio, followed by vortexing, centrifugation, and incubation at 30 °C for 30 min. Fresh $NaBH_4$ (10 mM) was then added to reduce Ag^+, and the reaction was allowed to proceed at 4 °C for at least 15 h. For hairpin constructs, samples were pre-heated to 95 °C for 2 min and snap-cooled on ice for 2 min prior to AgNC formation, followed by addition of $NaBH_4$ at the same concentration as $AgNO_3$. Excess reagents were removed using 3 kDa centrifugal filters with repeated washing in 4 mM NH_4OAc, and purified DNA-AgNCs were quantified by UV absorbance at 260 nm and diluted to the desired working concentrations.

Atomic Force Microscopy (AFM). All samples for AFM imaging were prepared by depositing DNA structures onto freshly cleaved mica modified with 1-(3- aminoropyl) silatrane (APS) according to previously described protocol [1, 2]. Briefly, 5 μL of sample solutions at various concentrations were deposited onto the modified mica and incubated for 2 min. Unbound nucleic acids and excess salts were removed by washing the surface twice with 50 μL of deionized water, after which the mica was dried under a gentle stream of argon gas. AFM imaging was performed using a MultiMode AFM Nanoscope V system (Bruker Instruments, Santa Barbara, CA) operated in PeakForce tapping mode with MSNL-E or MSNL-F probes from Bruker. PeakForce parameters, including amplitude, frequency, and setpoint were optimized to maintain peak interaction forces below 70 pN providing high-resolution imaging. AFM images were processed and analyzed using open-source software package Gwyddion.

Emission spectra. DNA-AgNC hairpins, Rings 6-IN, 6-OUT, and 12-IN-OUT were prepared at 14 μM AgNCs. Approximately 24 h after reduction, AgNC samples were transferred into a quartz cuvette, and emission scans were measured using a FluoroMax Plus spectrofluorometer (HORIBA). Excitation was set to 530 nm, and emission was measured at 636 nm. The excitation and emission slit widths were set at 1 nm and 3 nm, respectively.

3D fluorescence measurements. DNA-AgNC hairpins, Rings 6-IN, 6-OUT, and 12-IN-OUT were prepared at 14 μM AgNCs. Approximately 24 h after reduction, samples were transferred into a quartz cuvette and 3D excitation-emission scans were conducted using a FluoroMax Plus spectrofluorometer (HORIBA). Excitation and emission were measured from 500-700 nm and 500-800 nm, respectively, and monochromator slit widths were set at 3 nm.

Fluorescence aging. Fluorescence excitation-emission maps (EEMs) of DNA-AgNC hairpins, Rings 6-IN, 6-OUT, and 12-IN-OUT were collected at four time points: 1 day after reduction, and after 7, 14, and 21 days to evaluate aging and stability over time. Measurements were performed using a Duetta Fluorescence and Absorbance Spectrometer (HORIBA) with a sub-micro fluorometer cell (model 16.40F-Q-10, StarnaCells) at room temperature of ~22 °C. EEMs were recorded with 0.5 nm resolution, with emission scanned from 400 to 1000 nm and excitation from 300 to 750 nm. The resulting matrices were processed to generate 3D contour plots using MagicPlot Pro software. For aging analysis, emission intensities across full range were integrated at each excitation wavelength to produce spectrum-like traces shown in Figure 2D. This approach allowed for direct comparison of total fluorescence output and spectral evolution over time, providing clear view of changes in emission features during aging; specifically, it preserved the visibility of both the first and second emission peaks on the same trace.

Energy-dispersive X-ray spectroscopy. Samples of hairpin with 13 uM silver concentration were deposited dropwise on a boron EDX substrate and left to dry for 24 hours. Drops were sequentially added and left to dry for 5 days to deposit a sufficient amount of material for analysis. EDX data was collected on a Phenom XL G2 Desktop SEM.

Singlet oxygen measurements. DNA-AgNC hairpin and rings were prepared as described for excitation-emission matrices above. Each sample was prepared such that the concentration of silver was equivalent across the samples (13 uM silver). The samples were held inside a chilled storage container and transferred into a 1 cm pathlength quartz cuvette immediately prior to data collection. Data was obtained using a Horiba FluoroMax+ SpectroFluorometer with a liquid nitrogen cooled IR detector. The samples were irradiated at 565 nm light, and emission was collected between 800-1500 nm. Due to large scattering peaks, the emission intensity was determined by the integration of emission between 1200-1500 nm. Subsequent scans were conducted immediately following the conclusion of the preceding scan.

Quantum yield calculations. Data was collected using Horiba FluoroMax+ Spectrofluorometer. The AgNC's quantum yield could not be measured by integrating sphere due to their faint emission. Rhodamine B (99+% pure, laser grade, Thermo Scientific) was used for comparative quantum yield measurements as a standard. As the AgNC samples are kept in a buffer solution, the reference dye's quantum yield was first measured as 20.05% in identical conditions via integrating sphere.

Molecular dynamics simulations. The oxDNA systems were built by importing the all-atom pdb files and duck RIG-I pdb (PDB ID: 4A2P) into oxView[3]. For protein–RNA simulations, we used the anisotropic network model (ANM) for proteins, where each residue is reduced to a single site at its C-alpha position and residues within 13 Å are connected by harmonic springs, with the global spring constant fit to reproduce experimental C-α B-factors[4]. Duck RIG-I (PDB ID: 4A2P) was used as the structural template because the duck and human proteins share the same overall domain architecture and are close homologs (53% sequence identity), and duck RIG-I has been extensively characterized experimentally, including high-resolution crystal structures of the ligand-free, autorepressed, and RNA-bound, activated states[5]. Each simulation contained one nanoring with two RIG-I proteins positioned on opposite sides of the ring. Since the only interaction between protein residues and nucleotides is excluded volume, a spring potential was defined between two protein residues and two nucleotides for each RIG-I molecule. As the hybrid oxNA DNA/RNA model is not yet available within ANM-oxDNA, all nucleic acid components were modeled as RNA and simulated with the sequence-dependent RNANM model at 0.15 M monovalent salt in the NVT ensemble. We first performed a short Monte Carlo (MC) relaxation to remove steric clashes (10,000 steps, dt = 0.002, T = 0 °C, NVT) and then carried out production

molecular dynamics in the NVT ensemble for 1.1 × 10^7 steps with dt = 0.001 at T = 23 °C, using the Bussi thermostat (τ = 0.5). Simulations employed the oxDNA framework with RNANM interactions at 0.15 M monovalent salt, with configurations and energies saved every 5000 and 1000 steps, respectively. Post-processing was performed using the oxDNA Analysis Tools package[6]. All-atom RIG-I–RNA/DNA nanoring models were generated in BIOVIA Discovery Studio Visualizer for structural comparison only. The RIG-I model and FASTA sequence for the full structure were obtained from the protein data bank (PDB ID: 4A2W). Missing non-terminal residues were modeled with MODELLER[7] and missing terminal residues were estimated using AlphaFold[8].

Immune reporter cell assays. Human embryonic kidney (HEK) Lucia RIG-I cells (Invivogen) were maintained in 1× Dulbecco's Modified Eagle Medium (DMEM) supplemented with 10% heat-inactivated fetal bovine serum, 1% penicillin-streptomycin, 100 µg/mL Normocin, 30 µg/mL Blasticidin, and 100 µg/mL Zeocin. Cultures were incubated at 37 °C with 5% CO_2. For experiments, cells were plated at 20,000 cells per well in flat-bottom 96-well plates 24 h prior to transfection. RNA rings were incubated with Lipofectamine 2000 (Invitrogen) for 30 min at room temperature (0.375 µL per well). A short 5' triphosphate RNA hairpin (10 nM; Invivogen) served as a positive control for RIG-I activation. Twenty-four hours post-treatment, 50 µL of QUANTI-Luc (Invivogen) was added to 20 µL of cell supernatant, and luminescence was immediately measured using a Tecan Spark microplate reader.

Intracellular fluorescence measurements. Human embryonic kidney (HEK) 293-FT cells were maintained in Dulbecco's Modified Eagle Medium (DMEM) supplemented with 1% penicillin-streptomycin. Cell cultures were incubated at 37 °C, 5% CO_2. Cells were plated in a 24-well plate at 100,000 cells per 500 µL well and left to incubate overnight. Once cells were confirmed to be adhered to the wells, 50 µL of cell culture media was removed from the wells and replaced with 50 µL of AgNC ring treatments in fresh cell culture media. At 12, 24, and 48 h(?), cells were detached from the wells with 0.25% trypsin. Once cells were detached, trypsin was deactivated by addition of cell culture media, and cells were transferred via pipetting to glass microscopy slides.

Culture and maintenance of primary murine osteoblasts. Whole calvaria were collected from 2-3-day-old neonatal mice and cultured under differentiation conditions for 10 days, as previously described[9-12]. Differentiation was verified by alkaline phosphatase staining using a commercially available kit (Abcam) and confirmed by microscopy according to established protocols[10, 12].

S. aureus UAMS-1 propagation. *S. aureus* strain UAMS-1 was cultured overnight in Luria broth (LB) agar plates, then inoculated into LB media and incubated at 37ºC and 5% CO_2 overnight. Bacterial density and colony-forming units (CFU) were quantified using a Genespec3 spectrophotometer, as previously described[10-12] (MiraiBio Inc.).

S. aureus minimum inhibitory and bactericidal concentration determination. S. aureus cells (OD_{600} = 0.01) were treated with DNA-AgNC hairpins or AgNC rings in liquid culture and incubated for 18 h at 37ºC in a sterile 96-well plate. The MIC was selected as the lowest concentration associated with no visible growth or increase in optical density at 600 nm. Bacteria exposed to the MIC and lower were diluted in series, drop-plated on LB agar plates, and incubated overnight at 37ºC prior to colony counts. The MBC was determined at the lowest concentration that decreased bacterial viability by at least 99.9%, or a 3-log difference.

S. aureus fluorescence microscopy. S. aureus liquid culture started from a single colony was grown overnight in sterile LB media at 37 ºC. Planktonic bacteria were seeded with fresh LB media in 4 chambered glass bottom (35 mm) dishes (Cellvis) and left to adhere overnight at 37 ºC. Planktonic bacteria were washed off with 1X PBS, and early-stage *S. aureus* biofilms were treated with 6 μM DNA-AgNC hairpins. After 2 h incubation at 37ºC, *S. aureus* biofilms were washed with 1X PBS and stained with Sytox Deep Red (Invitrogen) for 15 min and room temperature prior to imaging at 63X with a Leica DMi8 fluorescence microscope using the Cy5 filter set (EX: 590-640 nm, EM: 662-738 nm).

S. aureus biofilm inhibition. AgNC hairpins (8 μM) and planktonic *S. aureus* (OD_{600} = 0.01) were incubated together in 4 chambered glass bottom (35 mm) dishes at 37ºC for 24 h in LB media. Planktonic bacteria were washed off with 1X PBS. Early-stage biofilms were stained with 0.1% crystal violet for 30 min at room temperature, washed once more with 1X PBS, and imaged at 63X with a Leica DMi8 fluorescence microscope.

Scanning electron microscopy. S. aureus was grown in sterile LB media on #1.5 glass coverslips in a 6-well microwell plate, treated with DNA-AgNC hairpins at a final concentration of 6 μM, and incubated for 24 h at 37 °C. Coverslips with adherent *S. aureus* were washed twice with PBS prior to fixing for 2 h in 2.5% glutaraldehyde, then washed once again with PBS before secondary fixation in 2% osmium tetroxide for 1 h. Biofilms were dried via increasing ethanol gradient, sputter-coated with 5 nm Au, and desiccated until SEM imaging via Phenom XL. Images were acquired at 0.1 Pa via backscattered electron detector.

S. aureus infection of primary murine osteoblasts. Primary murine osteoblasts were seeded at a density of 1 x 10^6 cells per well and infected with *S. aureus* at a multiplicity of infection (MOI) of 75 in antibiotic-free medium for 2 hours. Following infection, cells were washed with 1X phosphate-buffered saline (PBS) and the medium was replaced with fresh medium containing 1% penicillin-streptomycin to kill all extracellular bacteria. At 24 hours post-infection, cell supernatants and lysates were collected for analysis.

Transfection of primary murine osteoblasts. Primary murine osteoblasts were seeded at a density of 1 x 10^6 cells per well and infected with *S. aureus* at a multiplicity of infection (MOI) of 75 as described. Following infection, cells were transfected with either the AgNC hairpins or rings at 13 μM silver, or the RIG-I-specific ligand, 5' triphosphate RNA (5 μg/mL) using Lipofectamine 2000 in antibiotic-free medium for 2 hours. The medium was then replaced with fresh medium containing 1% penicillin-streptomycin. At 24 hours post-infection, cell supernatants and lysates were collected for analysis. To assess intracellular bacterial survival, osteoblasts were lysed with saponin, and contents were drop-plated on LB agar plates and incubated at 37ºC overnight for colony counting the following day.

siRNA treatment of primary murine osteoblasts. Osteoblasts were transfected with siRNA using RNAiMAX according to the manufacturer's guidelines. Silencer Select small interfering RNAs were used at a final concentration of 15 nM and incubated for 24 hours prior to AgNC transfection. siRNA targeted RIG-I (ThermoFisher Scientific; assay IDs s106376 and s106374) or a scrambled negative control siRNA (ThermoFisher Scientific; AM4613). Cell supernatants and whole-cell lysates were collected at the indicated time points.

Immunofluorescent microscopy of primary murine osteoblasts. Osteoblasts were transfected with AgNC hairpins or rings using Lipofectamine 2000. At 4 hours post-transfection, cells were fixed and stained for actin with Alexa Fluor 488 Phalloidin (ThermoFisher Scientific, A12379).

Nuclei were stained using DAPI (ThermoFisher Scientific, 62248). Images were acquired using a Leica DMi8 fluorescence microscope.

Enzyme-linked immunosorbent assay (ELISA). Specific capture ELISAs were performed to quantify IL-6 and IFN-β production by osteoblasts following *S. aureus* infection and transfection with AgNC hairpins or rings. Cytokine levels were measured using commercially available antibody pairs for IL-6 (BD Biosciences; 554400, 554402) and IFN-β (BD Biosciences; 519202, 508105). Recombinant IL-6 and IFN-β were used to generate standard curves, and sample protein concentrations were calculated based on absorbance values relative to the corresponding curve.

Immunoblot analysis. Whole-cell protein lysates were used for immunoblotting analysis to assess the expression levels of RIG-I. Detection was achieved using a rabbit monoclonal antibody against RIG-I (Cell Signaling, 3743S). β-actin (Cell Signaling, 4957S) was used as a loading control. Densitometric quantification was performed using Azure Spot Por (Azure Biosystems), and protein levels of RIG-I were normalized to β-actin.

Presentation and statistical analysis. Figures were prepared using Adobe Illustrator. Statistical analyses were performed as indicated in the figure legends using GraphPad Prism software. Data are presented as the mean of multiple biological replicates, with the sample size (N) specified for each dataset. Error bars represent mean ± standard deviation (SD) or standard error of the mean (SEM) as indicated in figure legends. Differences were considered statistically significant at $p < 0.05$ with a 95% confidence interval. In addition, physiologically meaningful changes, defined as twofold or greater deviations from baseline, were also monitored.

Supporting Figures

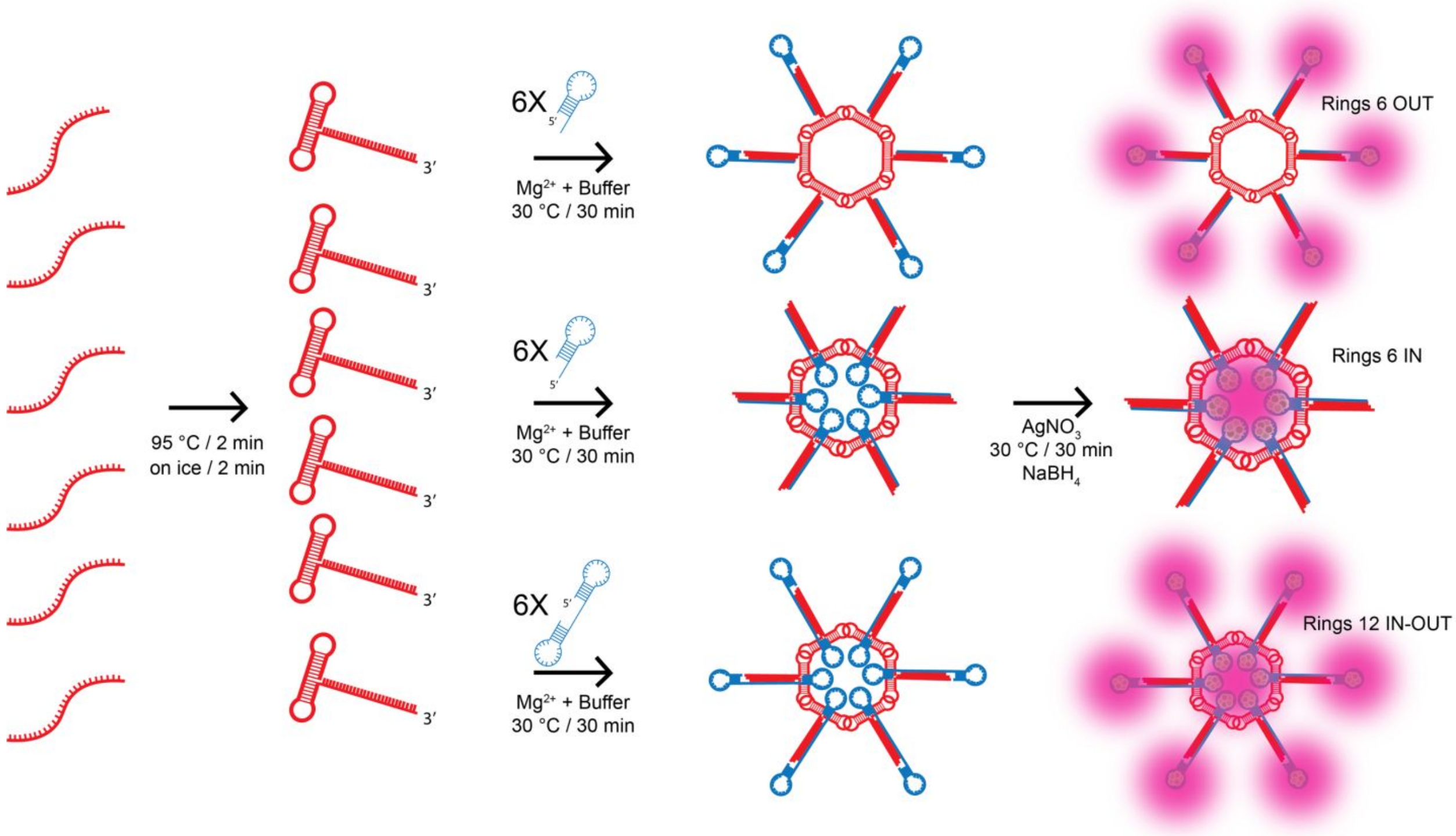

Figure S1: Schematic overview of AgNC rings assembly.

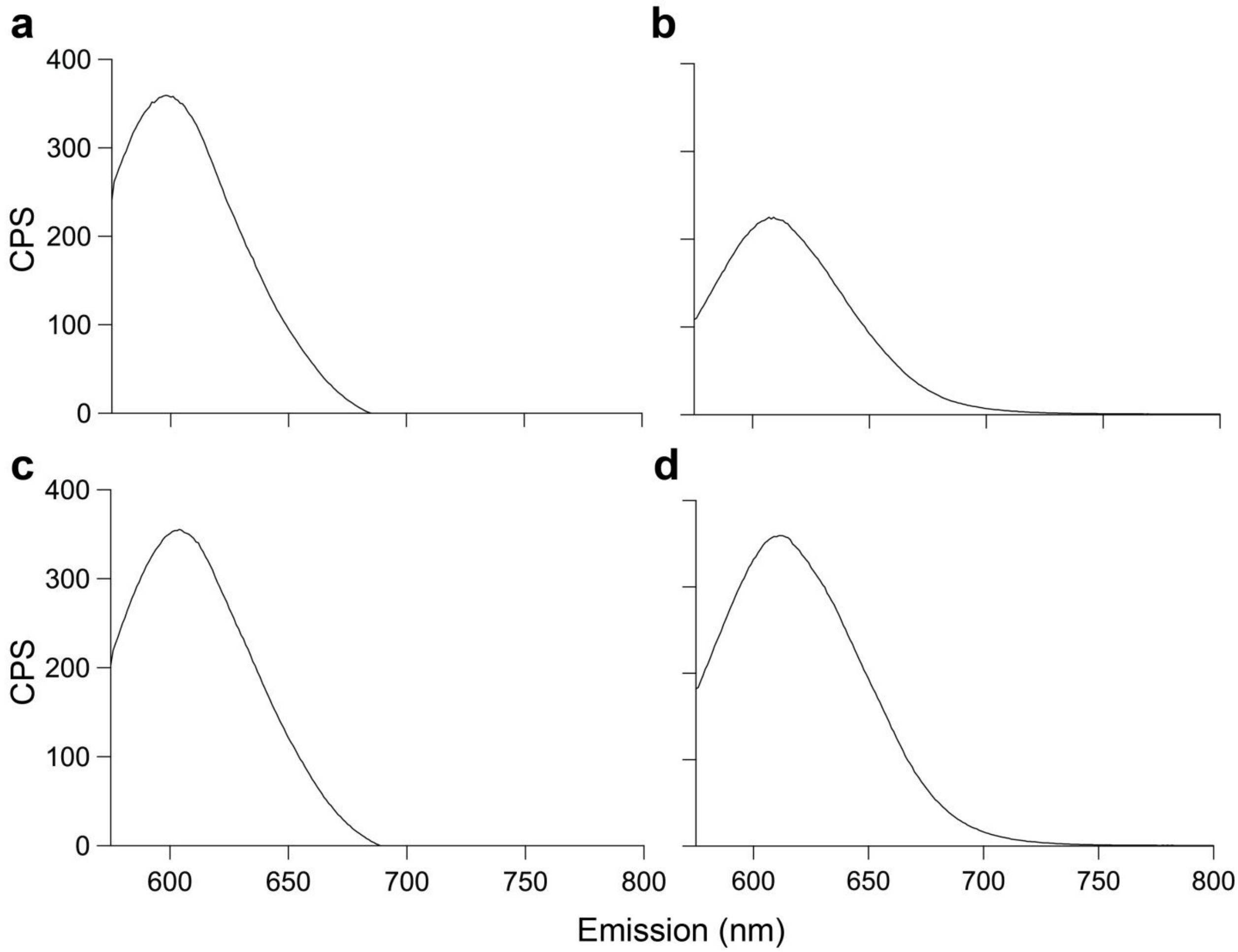

Figure S2: Representative emission spectra of (**a**) Rings 6 OUT, (**b**) Rings 6 IN, (**c**) Rings 12 IN-OUT, and (**d**) hairpins at 560 nm excitation.

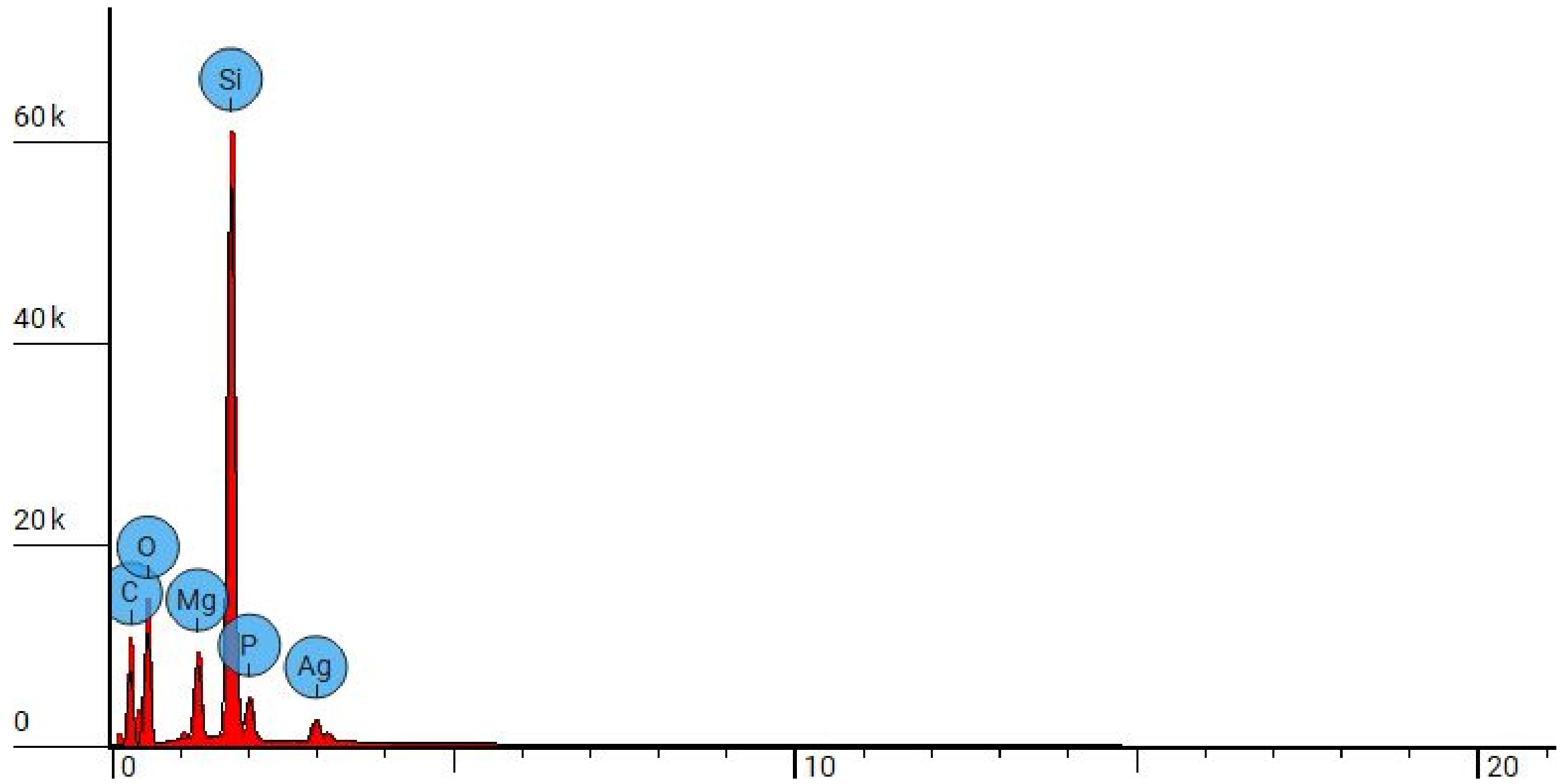

Figure S3: Representative EDX data for AgNC hairpin.

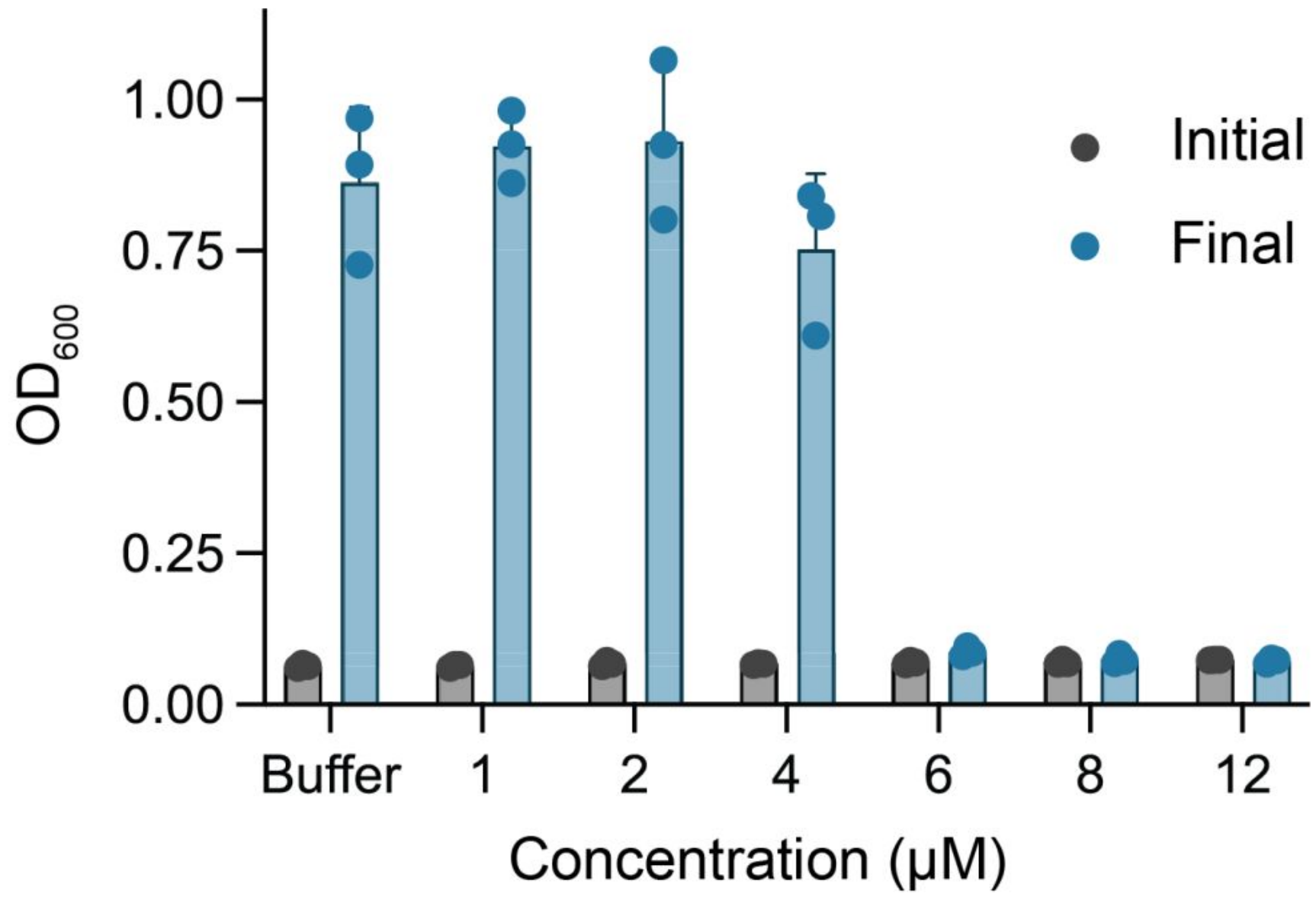

Figure S4: Optical density at 600 nm of planktonic *S. aureus* at time of hairpin treatment (initial) and after 18 h exposure (final).

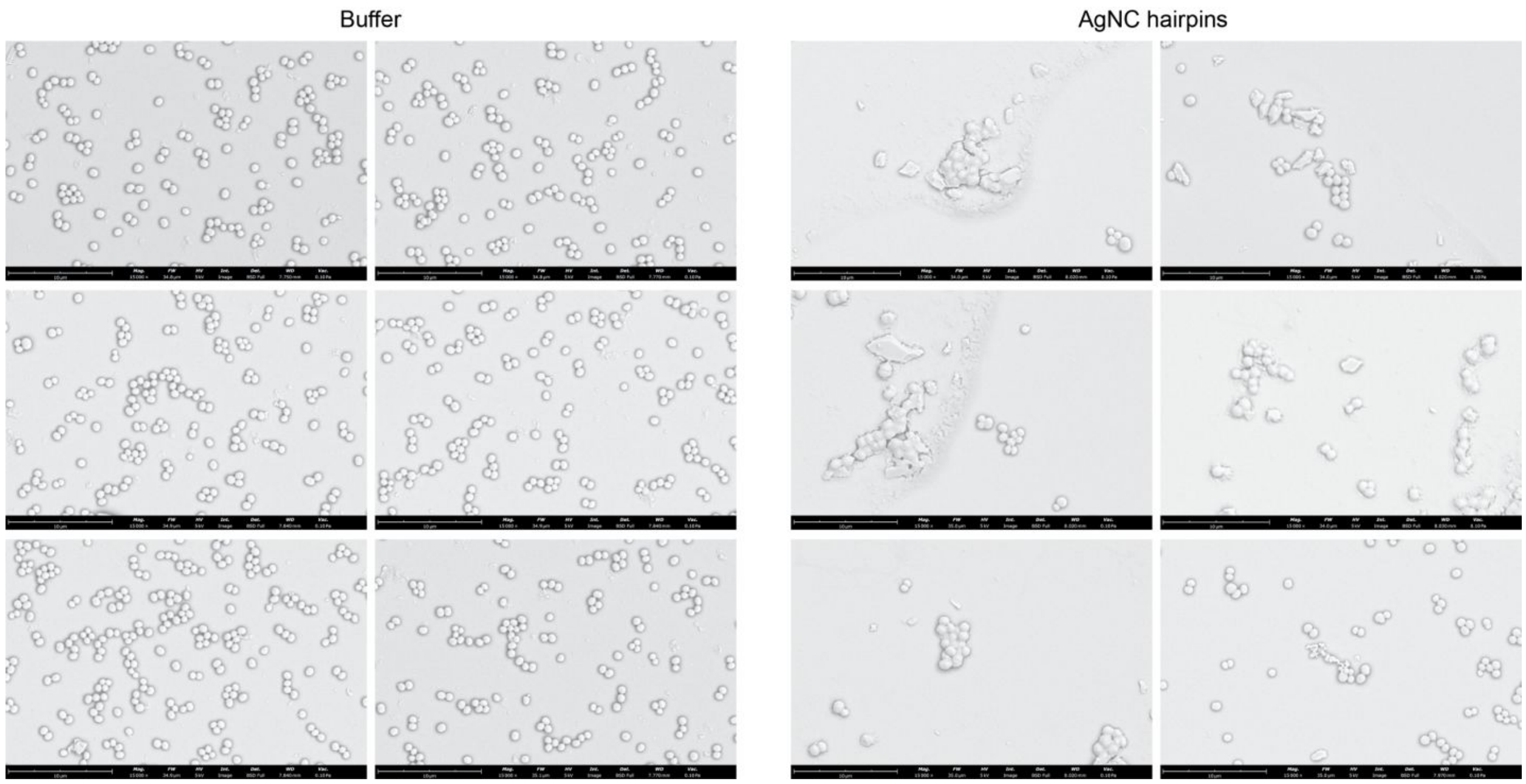

Figure S5: Representative SEM images of *S. aureus* following treatment with buffer or 6 µM DNA-AgNC hairpins.

a b

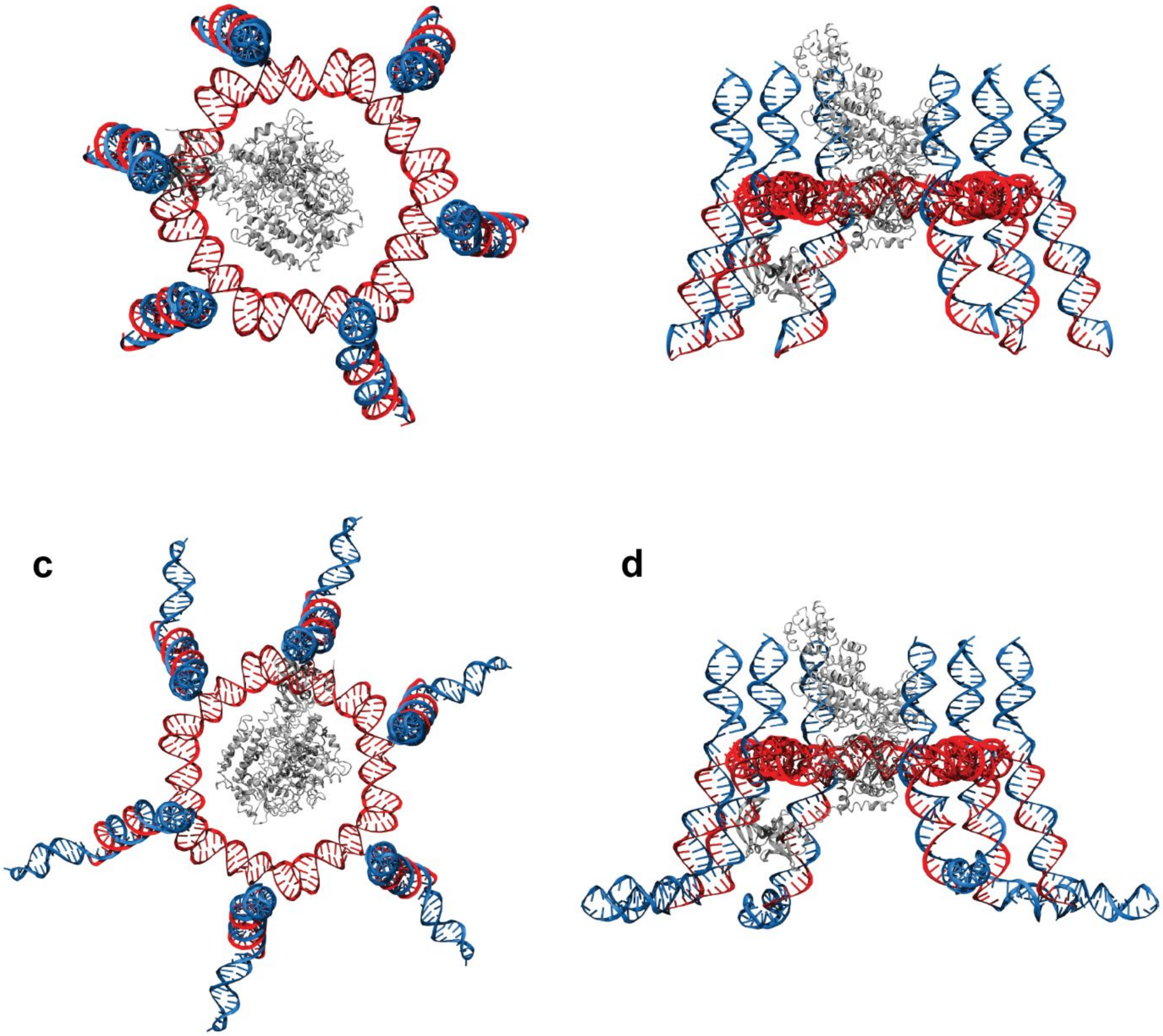

c d

Figure S6: All-atom models of Ring 6 IN from the top view (**a**) and side view (**b**). Ring 12 OUT top view (**c**) and side view (**d**).

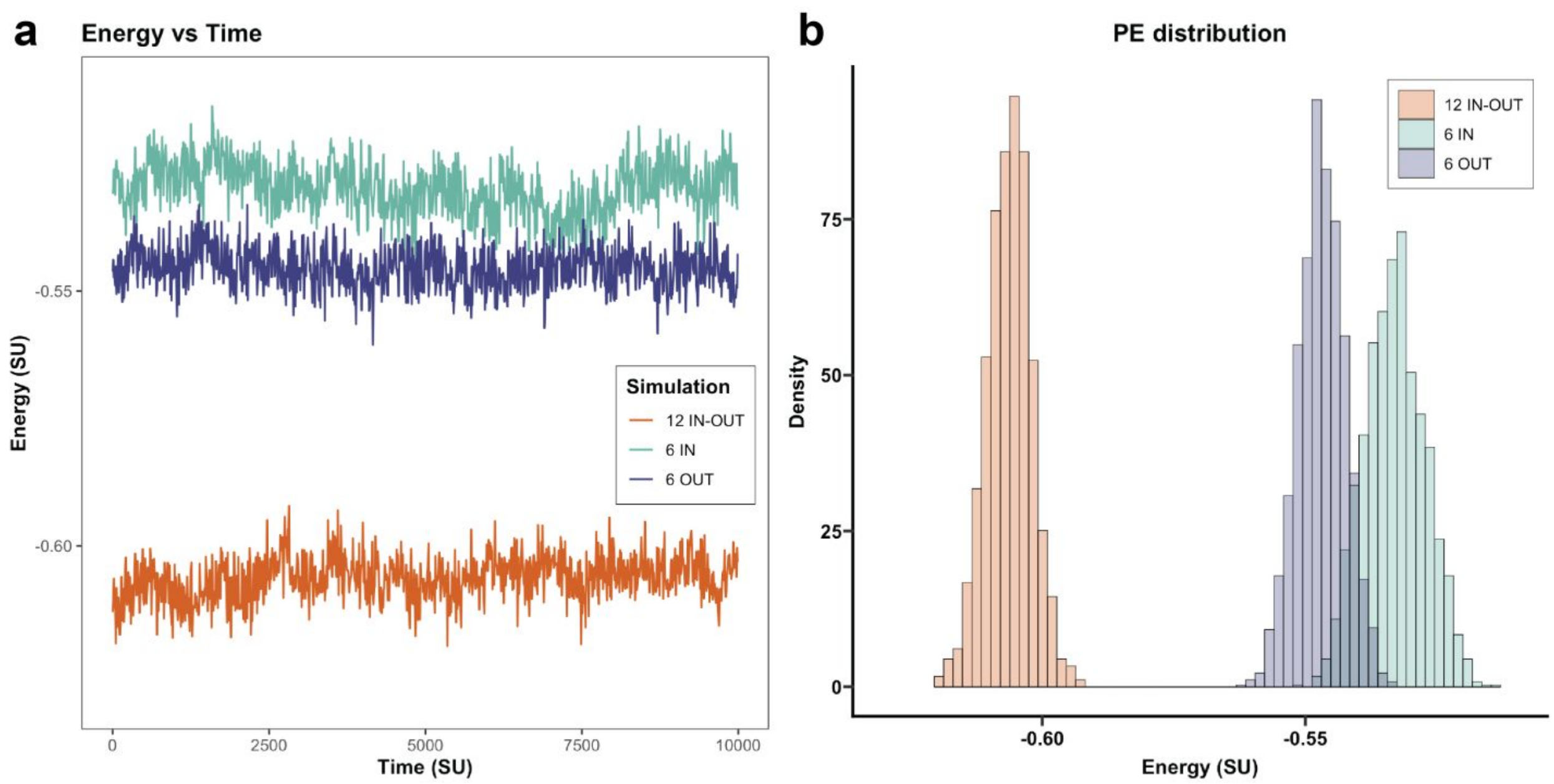

Figure S7. Potential energy (PE) from oxDNA simulations for Ring 6 OUT, Ring 6 IN, and Ring 12 IN–OUT shown as (**a**) time series and (**b**) energy distributions.

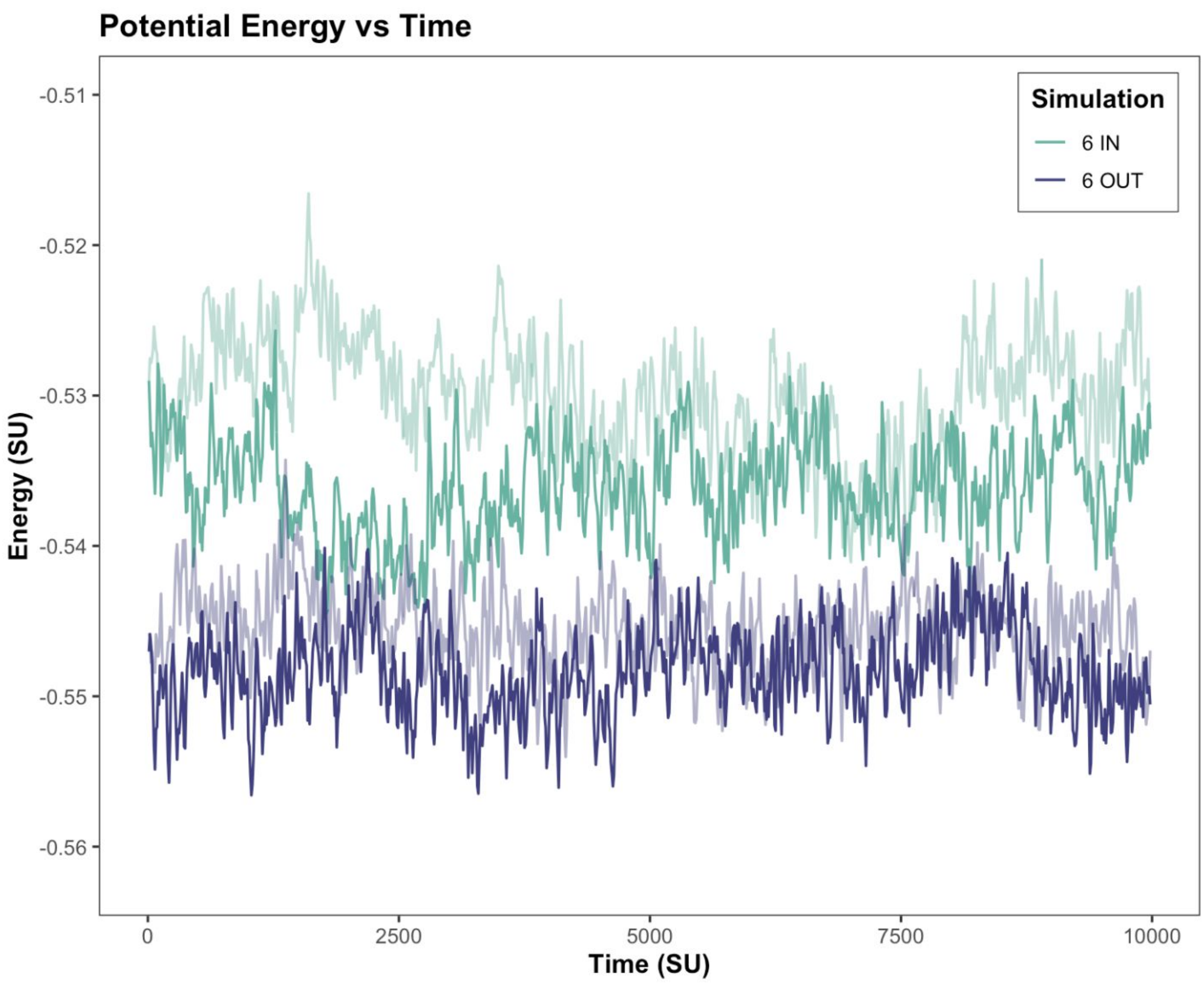

Figure S8. Potential energy (PE) during simulations of Ring 6 IN and Ring 6 OUT under two mutual trap forces (0.001 and 1.2 simulation units). Darker shading indicates the smaller force (0.001), and lighter shading indicates the larger force (1.2)

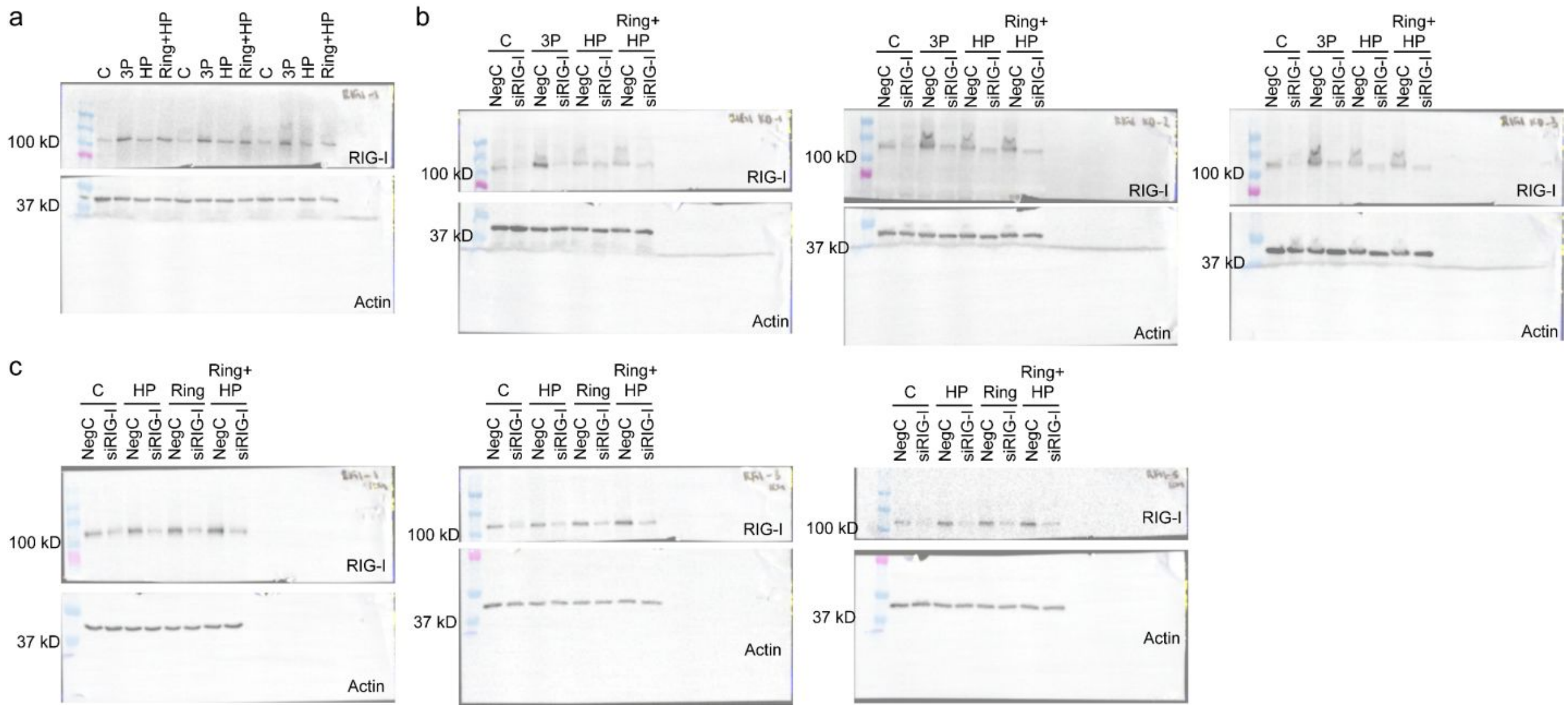

Figure S9: Primary murine osteoblasts were untreated (C) or transfected with 5' triphosphate RNA (3P; 5 μg/mL), free AgNC hairpin (HP; 13 μM silver), or Rings 6 OUT containing a single hairpin (Ring+HP; 13 μM silver). (**a**) Expression of RIG-I (102 kDa) at 24h assessed by immunoblot analysis and normalized to β-actin levels. Osteoblasts were treated with RIG-I-targeting siRNA (15 nM) prior to stimulation with 3P (5 μg/mL), HP (13 μM silver), Ring alone (0 μM silver), or Ring-HP (13 μM silver). (**b-c**) Expression of RIG-I (102 kDa) at 24h assessed by immunoblot analysis and normalized to β-actin levels.

Supporting Tables

Element Number	Element Symbol	Element Name	Atomic Conc. [%]	Weight Conc. [%]
6	C	Carbon	45.44	30.42
8	O	Oxygen	28.93	25.80
12	Mg	Magnesium	2.67	3.61
14	Si	Silicon	19.49	30.52
15	P	Phosphorus	2.62	4.52
47	Ag	Silver	0.85	5.12

Table S1: EDX elemental composition.

Material	Quantum Yield
Ref. (Rhodamine)	30.00%
Hairpin	2.66%
Rings 6-OUT	0.16%
Rings 6-IN	0.70%
Rings 12 IN-OUT	4.52%

Table S2: Fluorescence quantum yield calculations.

Supporting References

(1) Lushnikov, A.; Avila, Y.; Afonin, K.; Krasnoslobodtsev, A. Characterization of RNA Nanoparticles and Their Dynamic Properties Using Atomic Force Microscopy. Vol. 2709; 2023; pp 191-202.

(2) Shlyakhtenko, L. S.; Gall, A. A.; Filonov, A.; Cerovac, Z.; Lushnikov, A.; Lyubchenko, Y. L. Silatrane-based surface chemistry for immobilization of DNA, protein-DNA complexes and other biological materials. *Ultramicroscopy* **2003**, *97* (1-4), 279-287. DOI: 10.1016/s0304-3991(03)00053-6 From NLM.

(3) Bohlin, J.; Matthies, M.; Poppleton, E.; Procyk, J.; Mallya, A.; Yan, H.; Šulc, P. Design and simulation of DNA, RNA and hybrid protein–nucleic acid nanostructures with oxView. *Nature Protocols* **2022**, *17* (8), 1762-1788. DOI: 10.1038/s41596-022-00688-5.

(4) Procyk, J.; Poppleton, E.; Šulc, P. Coarse-grained nucleic acid–protein model for hybrid nanotechnology. *Soft Matter* **2021**, *17* (13), 3586-3593, 10.1039/D0SM01639J. DOI: 10.1039/D0SM01639J.

(5) Kowalinski, E.; Lunardi, T.; McCarthy, Andrew A.; Louber, J.; Brunel, J.; Grigorov, B.; Gerlier, D.; Cusack, S. Structural Basis for the Activation of Innate Immune Pattern-Recognition Receptor RIG-I by Viral RNA. *Cell* **2011**, *147* (2), 423-435. DOI: 10.1016/j.cell.2011.09.039 (acccessed 2026/02/21).

(6) Poppleton, E.; Bohlin, J.; Matthies, M.; Sharma, S.; Zhang, F.; Šulc, P. Design, optimization and analysis of large DNA and RNA nanostructures through interactive visualization, editing and molecular simulation. *Nucleic Acids Research* **2020**, *48* (12), e72-e72. DOI: 10.1093/nar/gkaa417 (acccessed 2/22/2026).

(7) Šali, A.; Blundell, T. L. Comparative Protein Modelling by Satisfaction of Spatial Restraints. *Journal of Molecular Biology* **1993**, *234* (3), 779-815. DOI: https://doi.org/10.1006/jmbi.1993.1626.

(8) Abramson, J.; Adler, J.; Dunger, J.; Evans, R.; Green, T.; Pritzel, A.; Ronneberger, O.; Willmore, L.; Ballard, A. J.; Bambrick, J.; et al. Accurate structure prediction of biomolecular interactions with AlphaFold 3. *Nature* **2024**, *630* (8016), 493-500. DOI: 10.1038/s41586-024-07487-w.

(9) Madrazo Denise, R.; Tranguch Susanne, L.; Marriott, I. Signaling via Toll-Like Receptor 5 Can Initiate Inflammatory Mediator Production by Murine Osteoblasts. *Infection and Immunity* **2003**, *71* (9), 5418-5421. DOI: 10.1128/iai.71.9.5418-5421.2003 (acccessed 2025/11/19).

(10) Sipprell Sophie, E.; Johnson, M. B.; Leach, W.; Suptela Samantha, R.; Marriott, I. Staphylococcus aureus Infection Induces the Production of the Neutrophil Chemoattractants CXCL1, CXCL2, CXCL3, CXCL5, CCL3, and CCL7 by Murine Osteoblasts. *Infection and Immunity* **2023**, *91* (4), e00014-00023. DOI: 10.1128/iai.00014-23 (acccessed 2025/11/19).

(11) Johnson, M. B.; Furr, K. H.; Suptela, S. R.; Leach, W.; Marriott, I. Induction of protective interferon-β responses in murine osteoblasts following Staphylococcus aureus infection. *Frontiers in Microbiology* **2022**, *Volume 13 - 2022*, Original Research. DOI: 10.3389/fmicb.2022.1066237.

(12) Johnson, M. B.; Suptela, S. R.; Sipprell, S. E.; Marriott, I. Substance P Exacerbates the Inflammatory and Pro-osteoclastogenic Responses of Murine Osteoclasts and Osteoblasts to Staphylococcus aureus. *Inflammation* **2023**, *46* (1), 256-269. DOI: 10.1007/s10753-022-01731-z From NLM.
